# Potential screening indicators for early diagnosis of NAFLD/MAFLD and liver fibrosis: Triglyceride glucose index–related parameters

**DOI:** 10.3389/fendo.2022.951689

**Published:** 2022-09-02

**Authors:** Yan Xue, Jiahui Xu, Man Li, Yueqiu Gao

**Affiliations:** ^1^ Laboratory of Cellular Immunity, Shuguang Hospital, Affiliated to Shanghai University of Traditional Chinese Medicine, Shanghai, China; ^2^ Department of Endocrinology, Shuguang Hospital Affiliated to Shanghai University of Traditional Chinese Medicine, Shanghai University of Traditional Chinese Medicine, Shanghai, China

**Keywords:** NAFLD, MAFLD, TyG index–related parameters, ROC curves, NHANES

## Abstract

**Importance:**

Homeostatic model assessment for insulin resistance (HOMA-IR) and triglyceride glucose (TyG) index–related parameters [TyG index, triglyceride glucose–waist circumference (TyG-WC), triglyceride glucose–waist-to-height ratio (TyG-WHtR), and triglyceride glucose–body mass index (TyG-BMI)] are gradually considered as convenient and alternative indicators for insulin resistance in various metabolic diseases, but the specific diagnostic capacity and the comparison of the parameters in non-alcoholic fatty liver disease (NAFLD), metabolic-associated fatty liver disease (MAFLD), and liver fibrosis remain uncertain.

**Objective:**

To comprehensively assess and compare the diagnostic accuracy of the above parameters in NAFLD, MAFLD, and liver fibrosis and identify the appropriate indicators.

**Methods:**

A total of 1,727 adults were enrolled from the 2017–2018 National Health and Nutrition Examination Surveys. Logistic regressions were used to identify the parameters significantly associated with NAFLD, MAFLD, and liver fibrosis; receiver operating characteristic (ROC) curves were used to evaluate and compare their diagnostic capacity. Subgroup analyses were conducted to validate the concordance, and the optimal cutoff values were determined according to the Youden’s indexes.

**Results:**

Significant differences were observed between quartile-stratified HOMA-IR and TyG index–related parameters across the NAFLD, MAFLD, and liver fibrosis (*P* < 0.05). All variables were significantly predictive of different disease states (*P* < 0.05). The top three AUC values are TyG-WC, TyG-WHtR, and TyG-BMI with AUCs of 0.815, 0.809, and 0.804 in NAFLD. The optimal cutoff values were 822.34, 4.94, and 237.77, respectively. Similar values and the same trend of the above three indexes could be observed in MAFLD and liver fibrosis. Subgroup analyses showed consistent results with the primary research, despite some heterogeneity.

**Conclusions:**

TyG-WC, TyG-WHtR, and TyG-BMI can be used for early screening of NAFLD and MAFLD. These three parameters and HOMA-IR were more suitable for assessing metabolic risks and monitoring disease progression in patients with NAFLD.

## Introduction

Non-alcoholic fatty liver disease (NAFLD), containing NAFL (hepatic steatosis alone) and NASH (with or without hepatic fibrosis and cirrhosis), is the most common liver metabolic disease in the world that affects approximately one-quarter of the global adult population (1, 2). The critical pathogenesis of NAFLD includes dysregulated hepatic glucose, lipid metabolism, insulin resistance (IR), and so on (3, 4). Based on the crucial impact of metabolic factors in NAFLD, researchers have proposed metabolic-associated fatty liver disease (MAFLD) as a more instructive medical diagnosis to describe this disease, reflecting current knowledge of NAFLD and associated metabolic dysfunction more accurately (2, 5). As the most common causes of cirrhosis, hepatocellular carcinoma (HCC), and the leading indication for liver transplants, the increased mortality and enormous economic burden in different countries caused by NAFLD caught everyone’s attention (6–8). It is essential to conduct early identification of NAFLD to reduce the various associated risks.

As the gold standard for diagnosing NAFLD, histopathological examination of liver biopsy has various limitations, such as invasiveness, poor acceptability, and higher cost (9). Therefore, it is urgent to explore noninvasive methods to diagnose NAFLD. The combination of serum markers and other indicators is valuable for screening diseases due to their convenience, low cost, and accuracy of diagnosis (10, 11). IR is involved in the pathogenesis and progression of NAFLD (4, 12). Homeostatic model assessment for IR (HOMA-IR), a valuable indicator for assessing IR, is widely used in IR-related disorders and is the diagnostic standard for MAFLD (2, 13). Triglyceride glucose (TyG) index–related parameters, including the TyG index, triglyceride glucose–body mass index (TyG-BMI), triglyceride glucose–waist-to-height ratio (TyG-WHtR), and triglyceride glucose–waist circumference (TyG-WC), were reported as credible and straightforward surrogate markers of IR as well as has significant value in metabolic-related diseases, including NAFLD (14, 15).

Some studies have suggested the relationship between NAFLD and TyG index–related parameters, HOMA-IR (14, 16). However, the role of the above parameters in different subgroups of the NAFLD population still needs to be further evaluated in a larger populace. Furthermore, diagnostic criteria of MAFLD incorporate numerous metabolic indicators; the ability of these parameters to access MAFLD and liver fibrosis remains a research gap. In the 2017–2018 National Health and Nutrition Examination Surveys (NHANES) cycle, examination data included the results of elastography using vibration-controlled transient elastography (VCTE) (17). VCTE is a widely used detection technology to quantify the severity of hepatic steatosis through controlled attenuation parameter (CAP; dB/m) score and degree of fibrosis through liver stiffness measurement (LSM; kPa) (18, 19). In this study, we try to explore and compare the value of these parameters (HOMA-IR, TyG index, TyG-WC, TyG-WHtR, and TyG-BMI) for predicting NAFLD, MAFLD, and liver fibrosis in the general adult population and the subgroups by using the data of VCTE.

## Methods

### Study design and population

**Figure 1:**
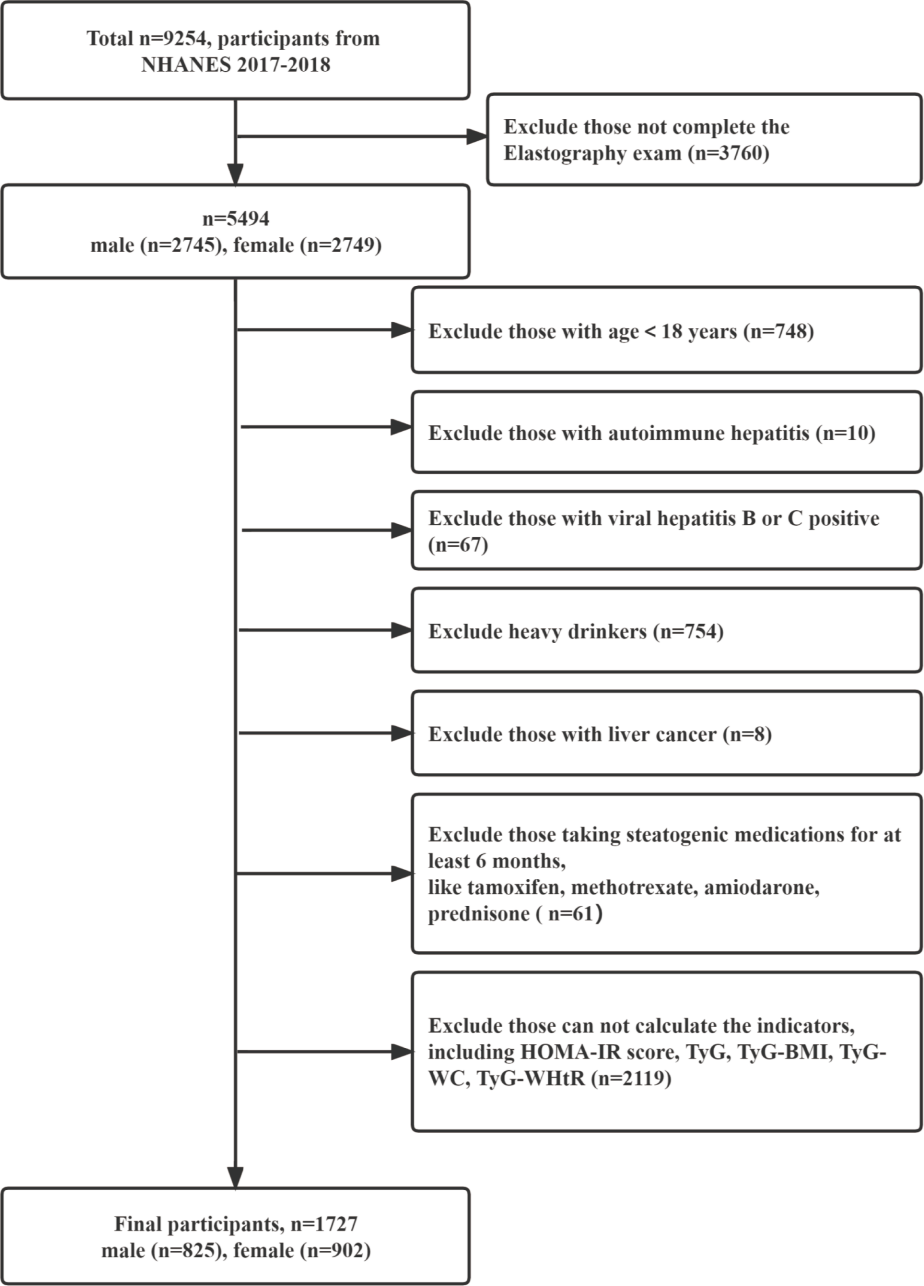
Flow chart of subject inclusion and exclusion in the 2017–2018 U.S. National Health and Nutrition Examination Survey.

This cross-sectional analysis used the 2017–2018 U.S. NHANES data because it has detailed VCTE examination data. NHANES was conducted with approval by the National Center for Health Statistics Ethics Review Board, and all participants gave written informed consent. The data are credible and nationally representative. The information of 9,254 participants was obtained in 2017–2018 NHANES; we firstly selected 5,494 participants who completed the elastography exam (male = 2,745; female = 2,749). Then, we excluded those participants in the following order (1): with age =18 years, (2) with autoimmune hepatitis, (3) with viral hepatitis B or C positive, (4) with liver cancer; (5) heavy drinkers [consumed more than two (female) or three (male) standard alcoholic drinks per day on average], (6) taking steatogenic medications for at least 6 months, and (7) those cannot be calculated the indicators. Finally, 1,727 participants were included in this study (**Figure 1**).

### Laboratory tests and clinical data

All of the variables were obtained from the original database and details are presented in online supplement information (20). Definitions, including education level, the family income-poverty ratio (21, 22), current smokers, hypertension (23), diabetes (24, 25), and overweight/obesity are also described in online supplement information. The parameters were defined or calculated as follows: waist-to-height ratio (WHtR) is defined as the waist circumference (WC) divided by the body height (26); HOMA-IR = insulin (μU/ml) × fasting glucose (mmol/L)/22.5 (13); TyG index = ln (fasting triglyceride [mg/dl] × fasting glucose [mg/dl]/2) (27); TyG-WHtR = TyG × WHtR (15); TyG-BMI = TyG index × BMI (28); TyG-WC = TyG × WC (28).

### The definition of NAFLD, MAFLD, and liver fibrosis

We use a median CAP ≥ 274 dB/m to define hepatic steatosis, and median LSM ≥ 7.0 kPa and LSM ≥ 8.2 kPa indicated the presence of liver fibrosis and moderate-to-advanced fibrosis (F≥F2), respectively (19, 29). NAFLD was diagnosed as the presence of hepatic steatosis in the absence of excessive alcohol consumption or other chronic liver diseases (18, 30), and MAFLD was defined as the presence of hepatic steatosis with one or more metabolic abnormalities referring to the new “positive’’ criteria (details are available in the Supplementary Material) (2, 31).

### Statistical analysis

All statistical analyses were conducted using IBM SPSS Statistics for Windows (version 25.0), MedCalc software (version 20.027), and the software GraphPad Prism (version 8.0.2). Data were described according to NAFLD and MAFLD status. Continuous variables that follow a normal distribution were described as means ± SD and analyzed by Student’s t-test. Non-normally distributed continuous variables were expressed as the median and interquartile range M (Q1–Q3) and analyzed by Mann-Whitney U-test. Frequency and proportions [n (%)] and the chi-square test represent and compare categorical variables. We assumed that these data were missing at random and used multiple imputations of missing adjusted variables (BP, PIR, and education level) by SPSS. Five imputed datasets were generated and merged to obtain the general result. The variance inflation factor (VIF) was used to evaluate multicollinearity in the multivariate analysis based on the intercorrelation between variables. Then, we transformed the variables (age, HOMA-IR, and TyG index–related parameters) into quartiles and conducted binary multivariable logistic regression with forwarding selection to explore the association between these parameters and NAFLD/MAFLD/liver fibrosis. Furthermore, the predictive values of the HOMA-IR and TyG index–related parameters (as continuous variables) were evaluated and compared using the receiver operating characteristic (ROC) curve analysis among the entire population as well as in subgroups of people. The value of the parameters corresponding to a maximum value of the Youden’s index [YI, max (J = sensitivity + specificity − 1)] was considered the ideal associated criterion (32). All tests were two-tailed, and *P* < 0.05 was considered statistically significant.

## Results

### Baseline characteristic

A total of 1,727 participants were enrolled in the final analyses. The clinical and biochemical characteristics of the participants were exhibited based on the status of NAFLD and MAFLD. Significant differences were observed between the four groups (NAFLD vs. non-NAFLD, MAFLD vs.. non-MAFLD, *P <* 0.05). The participants with NAFLD (n = 737, 42.68%) and MAFLD (n = 718, 41.58%) had higher BMI, WC, HOMA-IR, and TyG index–related parameters; also, older, male, without current smoking status, with diabetes, with hypertension, and overweight/obese people were more likely to develop NAFLD or MAFLD. A total of 170 (23.07%) patients had liver fibrosis among the population of NAFLD, and 166 (23.12%) people were diagnosed with MAFLD. Details are shown in **Table 1** and **Supplement Table 1**.

**Table 1:** Baseline clinical and biochemical characteristics of the participants.

Variables	Total, n = 1,727n (%) or M (Q1–Q3)	Non-NAFLD, n = 990n (%) or M (Q1–Q3)	NAFLD, n = 737n (%) or M (Q1–Q3)	*P*-value^*^
^a^Age (years)	53.00 (36.00–65.00)	47.50 (31.75–63.00)	57.00 (43.50–67.00)	<0.001
<36	429 (24.80)	320 (32.30)	109 (14.80)	<0.001
≥36, ≤53	441 (25.50)	247 (24.90)	194 (26.30)	
>53, ≤65	439 (25.40)	212 (21.40)	227 (30.80)	
>65	418 (24.20)	211 (21.30)	207 (28.10)	
Gender				0.003
Male	825 (47.80)	442 (44.60)	383 (52.00)	
Female	902 (52.20)	548 (55.40)	354 (48.00)	
PIR	1524 (88.24)			0.898
<1.0	264 (17.50)	147 (17.10)	117 (18.00)	
≤1.0, <4.0	823 (54.60)	471 (54.80)	352 (54.20)	
≥4.0	421 (27.90)	241 (28.10)	180 (27.70)	
Education level				0.520
Less than high school	277 (19.30)	153 (18.40)	124 (20.60)	
High school graduate or equivalent	340 (23.70)	196 (23.60)	144 (23.90)	
College or above	817 (57.00)	483 (58.10)	334 (55.50)	
Smoking	241 (14.00)	154 (15.60)	87 (11.80)	0.026
Diabetes	401 (23.20)	127 (12.80)	274 (37.20)	<0.001
Hypertension	751 (44.40)	346 (35.90)	405 (55.90)	<0.001
^b^Overweight or obesity	1230 (71.20)	563 (56.90)	667 (90.50)	<0.001
Variables	Total, n = 1,727 n (%) or M (Q1–Q3)	Non-NAFLD, n = 990 n (%) or M (Q1–Q3)	NAFLD, n = 737 n (%) or M (Q1–Q3)	*P*-value^*^
Weight (kg)	77.60 (65.80–92.50)	71.40 (60.88–83.70)	86.50 (75.05–102.75)	<0.001
Height (cm)	165.70 (58.70–173.40)	165.70(158.60–173.40)	165.70 (158.75–173.45)	0.538
WC (cm)	97.70 (87.20–109.60)	91.00 (82.20–101.20)	106.70 (97.30–117.50)	<0.001
WHtR	0.59 (0.53–0.66)	0.55 (0.49–0.61)	0.64 (0.59–0.70)	<0.001
BMI (kg/m^2^)	28.00 (24.40–32.80)	25.80 (23.00–29.40)	31.30 (27.80–35.90)	<0.001
FPG (mg/dl)	104.00 (97.00–115.00)	100.00 (95.00–108.00)	111.00 (101.00–128.00)	<0.001
Insulin (uIU/ml)	8.44 (5.37–13.58)	6.57 (4.35–9.78)	12.27 (8.05–19.07)	<0.001
ALT (IU/L)	17.00 (13.00–25.00)	15.00 (12.00–21.00)	21.00 (15.00–30.00)	<0.001
AST (IU/L)	19.00 (16.00–23.00)	18.00 (15.00–22.00)	20.00 (16.00–25.00)	<0.001
GGT (IU/L)	20.00 (14.00–30.00)	18.00 (13.00–25.00)	25.00 (18.00–38.00)	<0.001
HDL (mg/dl)	51.00 (43.00–61.00)	55.00 (46.00–66.00)	47.00 (41.00–55.50)	<0.001
LDL (mg/dl)	108.00 (87.00–132.00)	106.00 (85.00–129.00)	112.00 (88.00–134.00)	0.031
TC (mg/dl)	182.00 (159.00–212.00)	180.00 (155.00–209.00)	186.00 (161.00–217.00)	0.011
TG (mg/dl)	92.00 (61.00–134.00)	74.00 (52.00–109.25)	115.00 (80.00–162.50)	<0.001
^a^TyG index	8.50 (8.02–8.96)	8.24 (7.88–8.66)	8.81 (8.42–9.24)	<0.001
<8.02	433 (25.10)	352 (35.60)	81 (11.00)	
≥8.02, <8.50	431 (25.00)	298 (30.10)	133 (18.00)	
≥8.5, <8.96	430 (24.90)	207 (20.90)	223 (30.30)	
≥8.96	433 (25.10)	133 (13.40)	300 (40.70	
^a^HOMA-IR score	2.24 (1.38–3.84)	1.67 (1.10–2.52)	3.61 (2.22–5.85)	<0.001
<1.38	432 (25.00)	373 (37.70)	59 (8.00)	
≥1.38, <2.24	432 (25.00)	306 (30.90)	126 (17.10)	
Variables	Total, n = 1,727 n (%) or M (Q1–Q3)	Non-NAFLD, n = 990 n (%) or M (Q1–Q3)	NAFLD, n = 737 n (%) or M (Q1–Q3)	*P*-value^*^
≥2.24, <3.84	432 (25.00)	213 (21.50)	219 (29.70)	
≥3.84	431 (25.00)	98 (9.90)	333 (45.20)	
^a^TyG-WHtR	5.05 (4.34–5.81)	4.57 (3.99–5.19)	5.70 (5.08–6.35)	<0.001
<4.34	433 (25.10)	395 (39.90)	38 (5.20)	
≥4.34, <5.05	430 (24.90)	295 (29.80)	135 (18.30)	
≥5.05, <5.81	432 (25.00)	203 (20.50)	229 (31.10)	
≥5.81	432 (25.00)	97 (9.80)	335 (45.50)	
^a^TyG-BMI	240.41 (203.68–287.39)	216.20 (184.29–249.33)	276.50 (242.85–323.78)	<0.001
<203.68	431 (25.00)	393 (39.70)	38 (5.20)	
≥203.68,<240.41	432 (25.00)	304 (30.70)	128 (17.40)	
≥240.41, <287.39	432 (25.00)	188 (19.00)	244 (33.10)	
≥287.39	432 (25.00)	105 (10.60)	327 (44.40)	
^a^TyG-WC	838.50 (720.71–959.81)	761.40 (663.79–859.10)	941.38(846.32–1057.23)	<0.001
<720.71	431 (25.00)	395 (39.90)	36 (4.90)	
≥720.71, <838.50	432 (25.00)	297 (30.00)	135 (18.30)	
≥838.50, <959.81	432 (25.00)	200 (20.20)	232 (31.50)	
≥959.81	432 (25.00)	98 (9.90)	334 (45.30)	

Data are presented as the median and interquartile range for non-parametric variables as well as frequency and proportions for categorical variables. The characteristics of the study subjects were analyzed according to NAFLD status using the Mann-Whitney U-test to compare continuous variables and the chi-square test for categorical variables.

a Variables divided by quartiles.

b Overweight or obesity was defined as BMI ≥ 25 kg/m^2^.

^∗^P-value for the NAFLD and non-NAFLD groups.

### Association between the presence of NAFLD/MAFLD/liver fibrosis and the parameters

**Figure 2:**
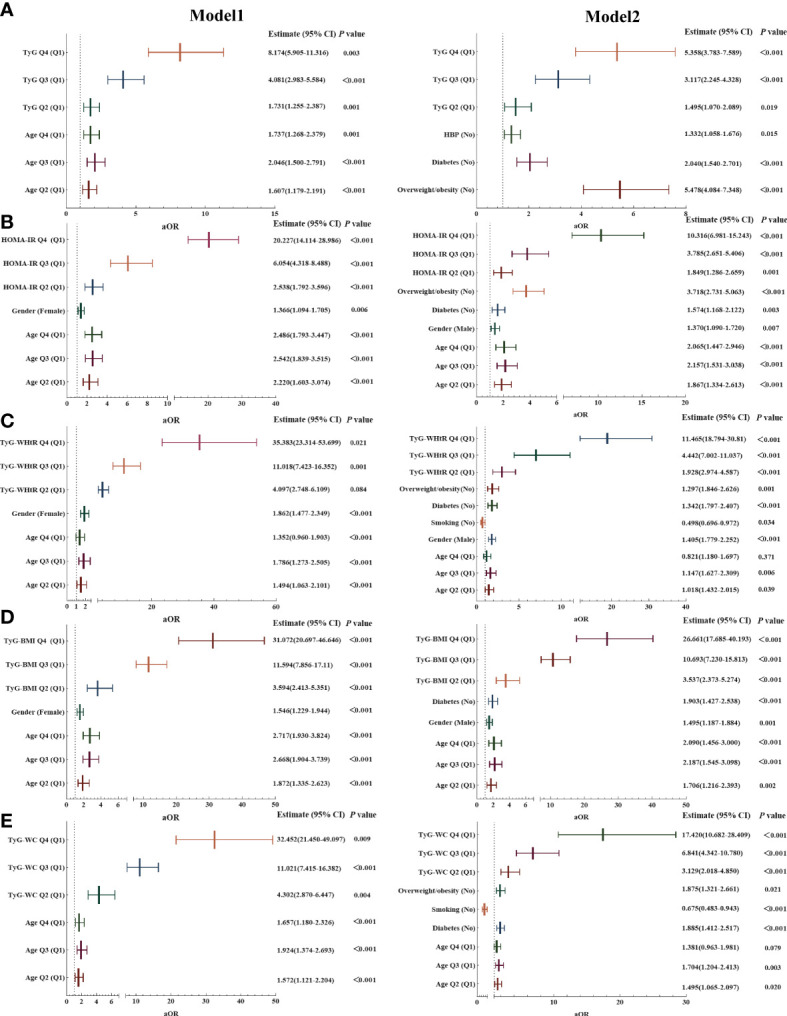
Association of the five parameters with non-alcoholic fatty liver disease (NAFLD). **(A)** Association of TyG index with NAFLD. **(B)** Association of HOMA-IR score with NAFLD. **(C)** Association of TyG-WHtR with NAFLD. **(D)** Association of TyG-BMI with NAFLD. **(E)** Association of TyG-WC with NAFLD.Model 1: Adjusted for age and gender.Model 2: Adjusted for age, gender, PIR, education level, smoking status, diabetes, hypertension, and obesity or overweight.Note: Q1–4 = quartiles 1–4; specific values are as follows:Age Q1 < 36 years, 36 ≥ Age Q2 ≤ 53 years, 53 > Age Q3 ≤ 65 years, Age Q4 > 65 years;TYG Q1 < 8.02, 8.02 ≥ TYG Q2 < 8.50, 8.5 ≥ TYG Q2 < 8.96, TYG Q4 ≥ 8.96;HOMA-IR Q1 < 1.38, 1.38 ≥ HOMA-IR Q2 < 2.24, 2.24 ≥ HOMA-IR Q2 < 3.84, HOMA-IR Q4 ≥ 3.84;TyG-WHtR Q1 < 4.34, 4.34 ≥ TyG-WHtR Q2 < 5.05, 5.05 ≥ TyG-WHtR Q2 < 5.81, TyG-WHtR Q4 ≥ 5.81;TyG-BMI Q1 < 203.68, 203.68 ≥ TyG-BMI Q2 < 240.41, 240.41 ≥ TyG-BMI Q2 < 287.39, TyG-BMI Q4 ≥ 287.39.TyG-WC Q1 < 720.71, 720.71 ≥ TyG-WC Q2 < 838.50, 838.50 ≥ TyG-WC Q2 < 959.81, TyG-WC Q4 ≥ 959.81.

To avoid multicollinearity, the model included the five parameters individually and excluded TC, TG, HDL, LDL, WC, WHtR, and weight (VIF ≥ 10). After adjusting for possible confounding factors, the comparisons of the five quartile-stratified parameters (HOMA-IR, TyG index, TyG-WC, TyG-WHtR, and TyG-BMI) across the presence of NAFLD showed significant differences between quartiles (**Table 1**, *P* < 0.001). The first model (Model 2), with minimal adjustment using age and gender, showed that the odds ratio (OR) was highest in TyG-WHtR, reaching 35.383 (95% CI 23.314–53.699) for the top quartile (Q4) compared to the first quartile (Q1) (*P* = 0.021) (**Figure 2**). The association was also significant after adjusting for age, gender, PIR, education level, smoking status, diabetes, hypertension, and obesity or overweight (the second model, Model 2), and TyG-BMI was the most prominent one in Model 2, reaching 26.661 (95% CI 17.685–40.193) for the top quartile (Q4) compared to the first quartile (*P* = 0.001) (**Figure 2**). Similar results were obtained in the analysis of MAFLD and liver fibrosis after the minimal adjustment. Higher levels of the five parameters were accompanied by raised risks for the high probability of MAFLD and developing liver fibrosis in NAFLD subjects; the details are shown in **Figure S1**.

### Predictive values of the parameters

To finally test the ability of these parameters to predict NAFLD, MAFLD, liver fibrosis, and moderate-to-advanced fibrosis, the ROC curves were calculated, and the AUCs were compared in the whole population as well as in each separated subgroups (grouped by age, gender, smoking status, diabetes, obesity/overweight, and hypertension).

#### ROC Curves and the AUC Values of the Five Parameters in Diagnosing Different Diseases Status of NAFLD Across All Populations

**Figure 3:**
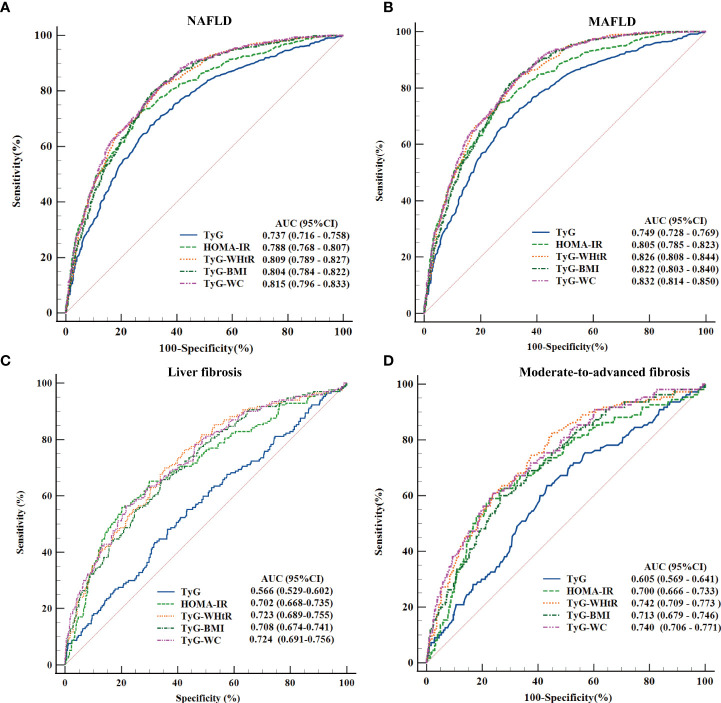
Receiver operating characteristic (ROC) curves and the area under the ROC curve (AUC) values of the five parameters (TyG index, HOMA-IR score, TyG-WHtR, TyG-BMI, and TyG-WC) in diagnosing NAFLD, metabolic-associated fatty liver disease (MAFLD), liver fibrosis, and moderate-to-advanced fibrosis. **(A)** Five parameters were assessed to identify NAFLD. **(B)** Five parameters were assessed to identify MAFLD. **(C)** Five parameters were assessed to identify liver fibrosis. **(D)** Five parameters were evaluated to identify moderate-to-advanced fibrosis.

Among the total population, all variables were significantly predictive of the different disease states (*P* < 0.05). The AUC values ranged from 0.737 to 0.832 in the diagnosis of NAFLD and MAFLD, whereas, in the population with NAFLD, the AUC values ranged from 0.566 to 0.742 in the diagnosis of liver fibrosis and moderate-to-advanced fibrosis. The top three AUC values in the comprehensive ranking were TyG-WC, TyG-WHtR, and TyG-BMI, with AUCs (95% CI) of 0.815 (0.796–0.833), 0.809 (0.789–0.827), and 0.804 (0.784–0.822) to predict NAFLD and 0.832 (0.814–0.850), 0.826 (0.808–0.844), and 0.822 (0.803–0.840) to predict MAFLD; detailed results are presented in **Figure 3**. According to the ROC curves and the Youden’s index, the optimal cutoff value of TyG-WC for NAFLD was 822.34 with 80.87% and 66.87% sensitivity and specificity and for moderate-to-advanced fibrosis was 1033.35 with 60.91% and 76.08%. The detailed results of TyG-WC, TyG-WHtR, and TyG-BMI were presented in **Supplement Table 2**.

To reconfirm the advantages of these parameters in predicting the risk of NAFLD and MAFLD, the AUCs of these three parameters were compared with WC, WHtR, BMI, FPG, TG, and insulin and the results fully affirm the excellent predictive values of TyG-WC, TyG-WHTR, and TyG-BMI (**Supplement Figure 2**). In addition, as illustrated in **Supplement Figure 3**, all indexes achieved statistical significance in distinguishing MAFLD from NAFLD, with the highest AUC values for HOMA-IR and the lowest for TyG index with AUC (95% CI) of 0.709 (0.678–0.740) vs.. 0.640 (0.607–0.672), *P* = 0.0139. The optimal cutoff value of the HOMA-IR score for distinguishing MAFLD from the NAFLD population was 2.35 with 74.79% and 60.71% sensitivity and specificity; more detailed values are given in **Supplemental Table 3** online.

### Subgroup analyses of the five parameters in diagnosing NAFLD, MAFLD, and liver fibrosis

**Figure 4:**
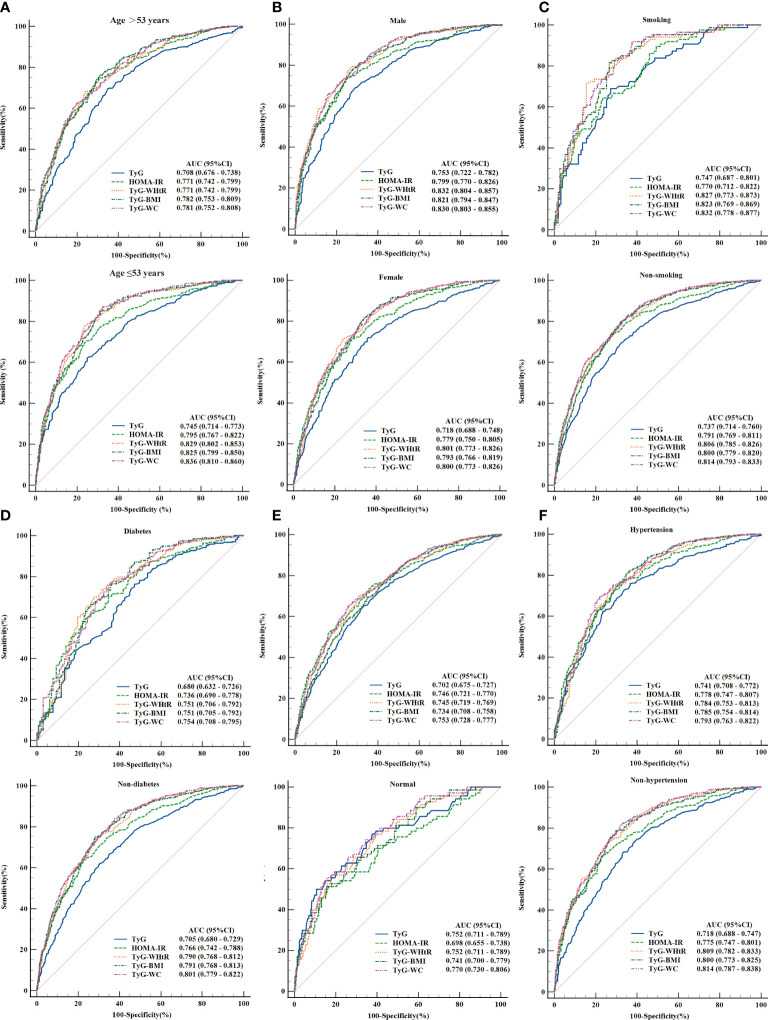
ROC curves and the area under the ROC curve (AUC) values of TyG index, HOMA-IR score, TyG-WHtR, TyG-BMI, and TyG-WC for NAFLD. **(A)** Subgroup analyses based on age. **(B)** Subgroup analyses based on gender. **(C)** Subgroup analyses based on smoking status. **(D)** Subgroup analyses based on diabetes. **(E)** Subgroup analyses based on overweight or obesity. **(F)** Subgroup analyses based on hypertension.

Furthermore, multiple subgroup analyses demonstrated limited statistical evidence of heterogeneity for some outcomes. The following points were observed:

(1) The analysis of NAFLD/MAFLD showed that higher AUC values were obtained in predicting MAFLD. TyG-WC, TyG-WHtR, and TyG-BMI remained the top three AUC values in the different subgroups, except for subgroups stratified based on hypertension and overweight/obesity. Moreover, the TyG index served as a valuable indicator in people without overweight/obesity with an AUC (95% CI) of 0.752 (0.711–0.789) in NAFLD and 0.862 (0.829–0.891) in MAFLD. All findings are summarised in **Figure 4** and **Supplement Figure 4**. Notably, when adjusted the BP threshold of the hypertensive subgroup to 130/85 mmHg (the criterion of MAFLD), the AUC values were improved (**Supplement Figure 5**).

(2) As displayed in **Supplement Figure 6**, some differences were obtained from the analyses of liver fibrosis. AUC values of HOMA-IR ranked the top three among the population of age ≥53 years, females, diabetic, and overweight/obese people, with AUC (95% CI) of 0.707 (0.662–0.750), 0.720 (0.670–0.766), 0.708 (0.651–0.761), and 0.703 (0.666–0.737), respectively. However, no significant difference was observed between HOMA-IR and the three parameters (TyG-WC, TyG-WHtR, and TyG-BMI) in all subgroups.

(3) The results of comparing the predictive ability of TyG-WC, TyG-WHtR, and TyG-BMI across the subgroups showed that the higher AUC value of TyG-WC was obtained among the people aged ≤53 years in NAFLD [AUC (95% CI) of (0.836 (0.810–0.860) vs.. 0.781 (0.752–0.808), *P* = 0.008]. At the same time, gender, smoking status, diabetes, hypertension, and obesity/overweight did not affect the diagnostic effect of TyG-WC (**Supplement Figure 7**). However, the subgroup analyses for MAFLD indicated that higher predictive values of TyG-WC emerged in the subgroups of non-diabetic (*P* < 0.05), non-overweight/non-obese (*P* < 0.05), and people aged ≤53 years (*P* < 0.05) (**Supplement Figure 8**). For the subgroup analysis of TyG-WC for liver fibrosis, no statistically significant differences were detected (*P* > 0.05) (**Supplement Figure 9**). Similar results were gained in TyG-WHtR and TyG-BMI (**Supplement Table 4**).

## Discussion

In this population-based study, we noticed a significant association between the TyG index, HOMA-IR, TyG-WHtR, TyG-BMI, and TyG-WC and the risks of NAFLD, MAFLD, as well as liver fibrosis in American adults. Advanced results from ROC curve analyses indicated that TyG-WC, TyG-WHtR, and TyG-BMI, especially TyG-WC, had better diagnostic values than the TyG index and HOMA-IR for predicting the above disease states, and these results were consistent with most of the subgroup analyses. Moreover, TyG-WC, TyG-WHtR, TyG-BMI and HOMA-IR could distinguish MAFLD from people with NAFLD. More detailed discussions of those observations are presented below.

The liver, a major metabolic organ, plays a vital role in regulating glucose and lipid metabolism. As crucial factors of NAFLD, IR and glucose/lipid dysmetabolism influence each other to promote the pathogenesis and progression of NAFLD (12, 33). Excessive intrahepatic triglyceride is the defining characteristic of NAFLD or MAFLD; IR causes excessive intrahepatic triglyceride by stimulating the *hepatic de novo* lipogenesis (DNL) and hepatic gluconeogenesis, etc.; the activated hepatic gluconeogenesis also increases glucose level (4, 34, 35). TyG index, calculated from fasting glucose and triglyceride levels, has been widely used as an important indicator of IR, predominantly peripheral and hepatic IR (27). Body mass index (BMI), WC, and WHtR are indexes for assessing obesity and are associated with the increased risk of IR, NAFLD, and other metabolic diseases (14, 36–41). The new parameters TyG-WC, TyG-WHtR, and TyG-BMI combine the above indexes and appear to be more reflective of IR status in NAFLD. Furthermore, the new parameters are more cost-effective and showed high diagnosis values in previous studies (15, 28). Therefore, the parameters above were introduced in our research for a further comprehensive assessment.

We assessed and compared the diagnostic accuracy of the five parameters (TyG index, HOMA-IR, TyG-WHtR, TyG-BMI, and TyG-WC). Consistent with previous research results, all the parameters could identify NAFLD and liver fibrosis in our study (14, 28, 42–44). Additionally, compared with TyG index and HOMA-IR, TyG-WHtR, TyG-BMI, and TyG-WC showed better identification ability for NAFLD, MAFLD, liver fibrosis, and moderate-to-advanced fibrosis. Plentiful studies have demonstrated that redefining NAFLD as MAFLD improves our awareness of predictors that increase the risk of death, including early identifying the potential metabolic complications (2, 45). The five parameters have higher predictive values for MAFLD than for NAFLD and a better ability to identify moderate-to-advanced fibrosis than liver fibrosis in our study. We also discovered that HOMA-IR showed the highest AUC value in distinguishing MAFLD among the NAFLD population. No significance was observed between the AUC values of TyG-WC, TyG-WHtR, TyG-BMI, and HOMA-IR, whereas a HOMA-IR score ≥ 2.5 is one of the diagnostic criteria for MAFLD. These results suggested that TyG-WC, TyG-WHtR, and TyG-BMI might be effective indicators of NAFLD with metabolic risks.

Further subgroup analyses supported these conclusions and displayed limited variability in some subgroups. The three indexes showed a better predictive value in identifying NAFLD and MAFLD among the younger population, consistent with previous results (14). Notably, the above study manifested higher AUC values among females, whereas our data showed no significant difference between the gender-grouped populations. Several potential factors could be responsible for this phenomenon. In the prior study conducted by Sheng et al., subjects diagnosed with diabetes or impaired fasting glucose at baseline were excluded (14), whereas females have higher insulin sensitivity and are less likely to develop obesity, IR, and diabetes (46). Interestingly, the three indexes showed higher diagnostic values in identifying MAFLD among non-diabetic and non-obese people, which might be related to the smaller number of MAFLD patients in the non-obese subgroup and larger prediabetic population in the non-diabetic subgroup.

In addition, some results in subgroups also showed valuable and suggestive implications. A study found that TyG-WHtR had the highest AUC for identifying fatty liver in the diabetic subgroup (15); our results demonstrated that TyG-WHtR, TyG-BMI, and TyG-WC had similar high diagnostic values to identify NAFLD and MAFLD among the population with diabetes through a larger sample population. A study enrolled 184 overweight/obese people (96 with and 88 without NAFLD, 30–65 years of age) suggested that TyG-BMI and TyG-WC had a good value in identifying NAFLD and liver fibrosis (47). Our study showed that TyG-WHtR, TyG-WC, and TyG-BMI are helpful indicators for NAFLD and MAFLD among the participants with or without obesity (overweight) and the non-overweight/non-obese people had a higher predictive value. Moreover, the TyG index was similar to the three parameters in the non-overweight/non-obese population. However, we failed to complete the subgroup analysis for liver fibrosis with the insufficient population after stratification. It should be noted that the poor predictive values of TYG-WHTR and TYG-BMI appeared in the non-hypertensive group in identifying MAFLD. Nonetheless, the results were reversed after adjusting the blood pressure threshold. This outcome indirectly verified the rationality of the blood pressure threshold in the diagnostic criteria of MAFLD.

The three indexes (TyG-WHtR, TyG-BMI, and TyG-WC) had high clinical diagnostic values in NAFLD, MAFLD, liver fibrosis, and moderate-to-advanced fibrosis. However, it is noteworthy that the three indexes showed higher AUC values but similar cutoff values in distinguishing moderate-to-advanced fibrosis compared with identifying liver fibrosis. These suggested that the above parameters might have better abilities to identify moderate-to-advanced fibrosis among the NAFLD population. It is wildly accepted that IR is closely associated with NAFLD and plays a vital role in the progression of NAFLD, including NASH (with liver fibrosis) (12). These three parameters are also considered novel indicators of IR and naturally capable of discriminative ability; the results remained consistent with the previous study (28, 48). Furthermore, research has shown that HOMA-IR was an independent predictor of advanced liver fibrosis in non-diabetic patients (43). The results in our subgroup analysis manifested that HOMA-IR had good diagnostic values for liver fibrosis among the population of aged >53 years, females, diabetic, and overweight/obese people. These stress the role of IR in the progression of NAFLD. Our study is the first to simultaneously explore and compare the diagnostic value of HOMA-IR and TyG-related parameters for liver fibrosis/moderate-to-advanced fibrosis. More importantly, we pointed out that TyG-WHtR, TyG-BMI, TyG-WC, and HOMA-IR could serve as excellent indicators for recognizing moderate-to-advanced fibrosis. The results are significant considering the association of moderate-to-advanced fibrosis with the risks of cirrhosis and the overall mortality in NAFLD.

Together, our observations include the following strengths. Firstly, we conducted stringent screening criteria to ensure the accurate diagnosis of NAFLD and applied the most recent standardized diagnostic criteria to define MAFLD. Therefore, our results are more representative and helpful in displaying the similarity and difference points between NAFLD and MAFLD. Secondly, our study comprehensively evaluated and compared the diagnostic values of HOMA-IR and TyG index–related parameters to identify different disease status (NAFLD, MAFLD, liver fibrosis, and moderate-to-advanced fibrosis) and conducted detailed subgroup analyses, which proved the stability and general applicability of TyG-WC, TyG-WHtR, and TyG-BMI. Thirdly, we thoroughly considered the importance of metabolic factors in the development and progression of NAFLD, the medical costs of NAFLD, as well as the necessity and feasibility of early detection in the population. The three indexes are easily accessible and could be used as valid diagnosis markers and plausible predictors of disease progression in a more efficient and cost-effective manner.

Three main limitations of this study were observed. First, this is a cross-sectional study limited to American adults. The diagnostic criteria of MAFLD and the definition of abnormal parameters, like BMI and WC, differ between the American and Asian standards, thus limiting the universality of our findings. In addition, we could not assess the longitudinal dynamic association between status changes in NAFLD and the level changing of these indicators. Second, we only evaluated liver fibrosis and moderate-to-advanced fibrosis to reveal the ability of these parameters to identify disease progression and did not evaluate the relationship between these parameters and NASH, though simple steatosis, which in the absence of NASH can have progression of biopsy-proven fibrosis and liver fibrosis, is a meaningful sign of disease progression (8, 49). Third, there were relatively small populations in specific subgroups, which likely created a bias and resulted in the inaccuracy of the diagnostic value of the five parameters in subpopulations.

## Conclusion

Our study demonstrated that TyG-WC, TyG-WHtR, and TyG-BMI were the most robust predictors to identify NAFLD, MAFLD, and moderate-to-advanced fibrosis in the U.S. adult population among the evaluation of all five indexes. In addition, HOMA-IR has a higher ability to distinguish MAFLD and liver fibrosis among NAFLD population. As the cheap and convenient indexes, TyG-WC, TyG-WHtR, and TyG-BMI were capable of guiding early intervention to control the process of NAFLD or MAFLD.

## Data availability statement

The original contributions presented in this study are included in the article, further inquiries can be directed to the authors.

## Ethics statement

Ethical review and approval was not required for the study on human participants in accordance with the local legislation and institutional requirements. The patients/participants provided their written informed consent to participate in this study.

## Author contributions

All authors made substantial contributions to the conception, design, analysis and interpretation of data. YX and JX collected the data and drafted the article, and YG and ML revised it critically for important intellectual content. All authors provided final approval of the version to be published and agreed to be accountable for all aspects of the work.

## Funding

This work was partly supported by National Natural Science Foundation of China (No. 81874436, 82074155); “Shuguang Program” supported by Shanghai Education Development Foundation and Shanghai Municipal Education Commission (No.18SG39, China); Program of Shanghai Academic/Technology Research Leader (No.20XD1423500, China); Clinical Research Plan of SHDC (No. SHDC2020CR3089B, SHDC2020CR1037B, China); Shanghai Frontier Research Base of Disease and Syndrome Biology of inflammatory cancer transformation (No.2021KJ03-12, China) ; Shanghai Collaborative Innovation Center of Industrial Transformation of Hospital TCM Preparation(China); In addition, this work was supported by Shanghai Key Laboratory of Traditional Chinese Medicine (20DZ2272200, China) and Key Laboratory of Liver and Kidney Diseases (Shanghai University of Traditional Chinese Medicine, China), Ministry of Education.

## Acknowledgments

We are very grateful to Hua Lv and Pinxian Huang for their guidance in data analysis.

## Conflict of interest

The authors declare that the research was conducted in the absence of any commercial or financial relationships that could be construed as a potential conflict of interest.

## Publisher’s note

All claims expressed in this article are solely those of the authors and do not necessarily represent those of their affiliated organizations, or those of the publisher, the editors and the reviewers. Any product that may be evaluated in this article, or claim that may be made by its manufacturer, is not guaranteed or endorsed by the publisher.

## Abbreviations

NAFLD, non-alcoholic fatty liver disease; MAFLD, metabolic-associated fatty liver disease; WC, waist circumference; WHtR, waist-to-height ratio; HOMA-IR, homeostatic model assessment for insulin resistance; TyG, triglyceride glucose index; TyG-BMI, triglyceride glucose–body mass index; TyG-WHtR, triglyceride glucose–waist-to-height ratio; TyG-WC, triglyceride glucose–waist circumference index; PIR, the family income poverty ratio; AST, aspartate aminotransferase; ALT, alanine transaminase; GGT, γ-glutamyl transferase; FPG, fasting plasma glucose; TC, total cholesterol; TG, triglyceride; LDL-C, low-density lipoprotein cholesterol; HDL-C, high-density lipoprotein cholesterol; aOR, adjusted OR; CI, confidence intervals; AUC, the area under the ROC curve; ROC, receiver operating characteristic.
