# Supplementary material for: Potential screening indicators for early diagnosis of NAFLD/MAFLD and liver fibrosis: Triglyceride glucose index–related parameters
Source: Front Endocrinol (Lausanne). 2022 Sep 2;13:951689. doi: 10.3389/fendo.2022.951689 (PMC9478620; doi:10.3389/fendo.2022.951689)
Supplement: Supplementary file 1 [file DataSheet_1.docx]

**Supplementary Material**

1. **Supplementary Data**

### Laboratory tests and clinical data

The following variables were obtained from the original database: 1) Demographic variables: age, sex, education level, the family income poverty ratio (PIR); 2) Examination variables: results of VCTE, height (cm), weight (kg), waist circumference (cm), blood pressure (BP), and body mass index (BMI) (calculated as weight in kilograms divided by height in meters squared, and then rounded to one decimal place kg/m2); 3) Laboratory Variables: alanine transaminase (ALT), aspartate aminotransferase (AST), γ-glutamyl transferase (GGT), albumin, fasting plasma glucose (FPG), insulin, total cholesterol (TC), triglyceride (TG), low-density lipoprotein cholesterol (LDL-C), high-density lipoprotein cholesterol (HDL-C), hepatitis B surface antigen (HBsAg), hepatitis C RNA (HCV-RNA), Hepatitis C Antibody (confirmed), High-Sensitivity C-Reactive Protein (hs-CRP) (mg/L); 4) Questionnaires: status of smoking, alcohol consumption, prescription medications use, history of high blood pressure or diabetes.

Definitions are described as follows. Education level was categorized into three categories: high, college or above; middle, high school graduate or equivalent; Low, less than high school. The family income-poverty ratio was categorized into high (≥ 4), middle (1-4), and low (< 1)^[21, 22]^. Current smokers were defined as those who smoked at least 100 cigarettes in life and smoked at the survey time. The mean of all available measurements was used to describe the SBP and DBP levels, and hypertension was defined as systolic blood pressure (SBP) ≥ 140 mmHg or diastolic blood pressure (DBP) ≥ 90 mmHg or self-reported use of antihypertensive medications^[23]^. Diabetes was defined as the presence of any of the following: previous diabetes diagnosis; use of anti-diabetic drugs (diabetic pills or/and insulin); the level of glycated hemoglobin A1c (HbA1c) ≥ 6.5%; a fasting blood glucose level of ≥7.0 mmol/l^[24, 25]^. Overweight or obesity is defined as a BMI ≥25 Kg/m^2^, while a healthy weight is BMI < 25 kg/m^2^.

**The definition of NAFLD, MAFLD, and liver fibrosis**

MAFLD was defined as the presence of hepatic steatosis with one or more of the following[[2, 31]:

1). Overweight or obese;

2). Diabetes mellitus;

3). All of the following items at least meet two metabolic abnormalities:

a. Waist circumference ≥102 cm in men and ≥88 cm in women;

b. Blood pressure ≥ 130/85 mmHg;

c. Triglycerides ≥ 1.70 mmol/L;

d. High-density lipoprotein cholesterol < 1.0 mmol/L for men and <1.3 mmol/L for women;

e. Prediabetes (i.e., fasting glucose levels 5.6 to 6.9 mmol/L, or HbA1c 5.7% to 6.4%;

f. HOMA-IR score ≥2.5;

g. Plasma high-sensitivity C-reactive protein level 2 mg/L.

**Sensitivity analyses**

Given the the close correlation between NAFLD and hyperlipidemia, we performed sensitivity analyses with 1007 individuals excluding subjects with hyperlipidemia (n = 720). This did not change the diagnostic abilities of the these indices (Supplemental Table 3).

**2.** **Supplementary Figure legends**

**Supplementary Figure 1**. Association between quartile of the five parameters and the risk of metabolic associated fatty liver disease and liver fibrosis, after adjustment for age and gender. (A) Association of TyG index with MAFLD. (B) Association of HOMA-IR score with MAFLD. (C) Association of TyG-WHtR with MAFLD. (D) Association of TyG-BMI with MAFLD. (E) Association of TyG-WC with MAFLD. (F) Association of TyG index with liver fibrosis. (G) Association of HOMA-IR score with liver fibrosis. (H) Association of TyG-WHtR with liver fibrosis. (I) Association of TyG-BMI with liver fibrosis. (J) Association of TyG-WC with liver fibrosis.

Note: Q1-4=quartile1-4; And specific values are as follows:

Age Q1 < 36 years, 36 ≥ Age Q2 ≤ 53 years, 53 > Age Q3 ≤ 65 years, Age Q4 > 65 years;

TYG Q1 < 8.02, 8.02 ≥ TYG Q2 < 8.50, 8.5 ≥ TYG Q2 < 8.96, TYG Q4 ≥ 8.96;

HOMA-IR Q1 < 1.38, 1.38 ≥ HOMA-IR Q2 < 2.24, 2.24 ≥ HOMA-IR Q2 < 3.84, HOMA-IR Q4 ≥ 3.84;

TyG-WHtR Q1 < 4.34, 4.34 ≥ TyG-WHtR Q2 < 5.05, 5.05 ≥ TyG-WHtR Q2 < 5.81, TyG-WHtR Q4 ≥ 5.81;

TyG-BMI Q1 < 203.68, 203.68 ≥ TyG-BMI Q2 < 240.41, 240.41 ≥ TyG-BMI Q2 < 287.39, TyG-BMI Q4 ≥ 287.39.

TyG-WC Q1 < 720.71, 720.71 ≥ TyG-WC Q2 < 838.50, 838.50 ≥ TyG-WC Q2 < 959.81, TyG-WC Q4 ≥ 959.81.**Supplementary Figure 2.** Comparing the predictive capabilities of TyG-WC, TyG-WHtR and TyG-BMI with other influencing factors. (A) Compared with WC, WHtR and BMI for NAFLD. (B) Compared with WC, WHtR and BMI for MAFLD. (C) Compared with FPG, TG and insulin for NAFLD. (D) Compared with FPG, TG and insulin for MAFLD.

**Supplementary Figure 3.** Receiver operating characteristic (ROC) curves and the area under the ROC curve (AUC) values of TyG index, HOMA-IR score, TyG-WHtR, TyG-BMI and TyG-WC to distinguish MAFLD from NAFLD.

**Supplementary Figure 4.** Receiver operating characteristic (ROC) curves and the area under the ROC curve (AUC) values of TyG index, HOMA-IR score, TyG-WHtR, TyG-BMI and TyG-WC for metabolic associated fatty liver disease (MAFLD). (A) Subgroup analyses based on age; (B) Subgroup analyses based on gender; (C) Subgroup analyses based on smoking status; (D) Subgroup analyses based on diabetes; (E) Subgroup analyses based on overweight or obesity; (F) Subgroup analyses based on hypertension.

**Supplementary Figure 5.** Comparing the area under the ROC curve (AUC) values of TyG-WHtR and TyG-BMI for MAFLD between the BP ≥ 130/85 mmHg and BP<130/85 mmHg subgroups. (A) Comparing the AUC values of TyG-WHtR; (B) Comparing the AUC values of TyG-BMI.

**Supplementary Figure 6.** Receiver operating characteristic (ROC) curves and the area under the ROC curve (AUC) values of TyG index, HOMA-IR score, TyG-WHtR, TyG-BMI and TyG-WC for liver fibrosis. (A) Subgroup analyses based on age; (B) Subgroup analyses based on gender; (C) Subgroup analyses based on smoking status; (D) Subgroup analyses based on diabetes; (E) Subgroup analyses based on overweight or obesity; (F) Subgroup analyses based on hypertension.

Note: We fail to [complete](javascript:;) the subgroups analyses with the insufficient non-obesity/ overweight subgroups population.

**Supplementary Figure 7.** Comparing the predictive capabilities of TyG-WC for NAFLD within different subgroups. (A) Subgroup analysis based on age; (B) Subgroup analysis based on gender; (C) Subgroup analysis based on smoking status; (D) Subgroup analysis based on diabetes; (E) Subgroup analysis based on overweight or obesity; (F) Subgroup analysis based on hypertension.

**Supplementary Figure 8.** Comparing the predictive capabilities of TyG-WC for MAFLD within different subgroups. (A) Subgroup analysis based on age; (B) Subgroup analysis based on gender; (C) Subgroup analysis based on smoking status; (D) Subgroup analysis based on diabetes; (E) Subgroup analysis based on overweight or obesity; (F) Subgroup analysis based on hypertension.

**Supplementary Figure 9.** Comparing the predictive capabilities of TyG-WC for liver fibrosis within different subgroups. (A) Subgroup analysis based on age; (B) Subgroup analysis based on gender; (C) Subgroup analysis based on smoking status; (D) Subgroup analysis based on diabetes; (E) Subgroup analysis based on hypertension.

Note: We fail to complete the subgroups analysis with the insufficient people without overweight or obese population.

**Supplementary Tables**

**Supplementary Table 1.** Baseline clinical and biochemical characteristics of the participants.

| Variables | Total, n=1727  n (%) or M (Q1-Q3) | Non-MAFLD, n=1009  n (%) or M (Q1-Q3) | | | MAFLD, n=718  n (%) or M (Q1-Q3) | *P* value^*^ |
| --- | --- | --- | --- | --- | --- | --- |
| Age (years) | 53.00 (36.00-65.00) | 37.00 (25.00-56.00) | | | 56.00 (39.00-66.00) | ＜0.001 |
| ＜36 | 429 (24.80) | 327 (32.40) | | | 102 (14.20) |  |
| ≥36, ≤53 | 441 (25.50) | 252 (25.00) | | | 189 (26.30) |  |
| ＞53, ≤65 | 439 (25.40) | 216 (21.40) | | | 223 (31.10) |  |
| ＞65 | 418 (24.20) | 214 (21.20) | | | 204 (28.40) |  |
| Gender |  |  | | |  | 0.002 |
| Male | 825 (47.80) | 451 (44.70) | | | 374 (52.10) |  |
| Female | 902 (52.20) | 558 (55.30) | | | 344 (47.90) |  |
| PIR | 1524 (88.24） |  | | |  | 0.725 |
| <1.0 | 264 (17.50) | 148 (16.90) | | | 116 (18.40) |  |
| ≤1.0, <4.0 | 823 (54.60) | 479 (54.70) | | | 344 (54.40) |  |
| ≥4.0 | 421 (27.90) | 249 (28.40) | | | 172 (27.20) |  |
| Education level |  |  | | |  | 0.446 |
| Less than high school | 277 (19.30) | 156 (18.30) | | | 121 (20.80) |  |
| High school graduate or equivalent | 340 (23.70) | 200 (23.50) | | | 140 (24.00) |  |
| College or above | 817 (57.00) | 495 (58.20) | | | 322 (55.20) |  |
| Smoking | 241 (14.00) | 156 (15.50) | | | 85 (11.80) | 0.032 |
| Diabetes | 401 (23.20) | 127 (12.60) | | | 274 (38.20) | ＜0.001 |
| Hypertension | 751 (44.40) | 349 (35.50) | | | 402 (56.9) | ＜0.001 |
| ^b^Overweight or obesity | 1230 (71.20) | 563 (55.80) | | | 667 (92.90) | ＜0.001 |
| Weight (kg) | 77.60 (65.80-92.50) | 60.15 (54.20-67.73） | | | 83.30 (71.60-97.00) | ＜0.001 |
| Variables | Total, n=1727  n (%) or M (Q1-Q3) | Non-MAFLD, n=1009  n (%) or M (Q1-Q3) | | | MAFLD, n=718  n (%) or M (Q1-Q3) | *P* value^*^ |
| Height (cm) | 165.70 (58.70-173.40) | 165.80 (158.88-173.53) | | | 165.70 (158.65-173.35) | 0.641 |
| WC (cm) | 97.70 (87.20-109.60) | 80.20 (75.08-85.60) | | | 102.30 (93.50-112.70) | ＜0.001 |
| WHtR | 0.59 (0.53-0.66) | 0.48 (0.45-0.52) | | | 0.62 (0.56-0.68) | ＜0.001 |
| BMI (kg/m2) | 28.00 (24.40-32.80) | 22.30 (20.40-23.80) | | | 29.70 (26.60-34.10) | ＜0.001 |
| FPG (mg/dL) | 104.00 (97.00-115.00) | 97.00 (93.00-102.25) | | | 106.00 (98.50-120.00) | ＜0.001 |
| Insulin (uIU/mL) | 8.44 (5.37-13.58) | 4.82 (3.32-6.82) | | | 9.77 (6.51-15.26) | ＜0.001 |
| ALT (IU/L) | 17.00 (13.00-25.00) | 14.00 (11.00-20.00) | | | 18.00 (13.00-26.00) | ＜0.001 |
| AST (IU/L) | 19.00 (16.00-23.00) | 18.00 (15.00-22.00) | | | 19.00 (16.00-24.00) | 0.017 |
| GGT (IU/L) | 20.00 (14.00-30.00) | 16.00 (11.75-21.00) | | | 22.00 (15.75-33.00) | ＜0.001 |
| HDL (mg/dL) | 51.00 (43.00-61.00) | 60.00 (51.75-71.00) | | | 49.00 (41.00-58.00) | ＜0.001 |
| LDL (mg/dL) | 108.00 (87.00-132.00) | 101.00 (81.75-123.00) | | | 111.00 (88.00-134.00) | ＜0.001 |
| TC (mg/dL) | 182.00 (159.00-212.00) | 176.00 (153.75-204.00) | | | 184.00 (160.00-214.00） | 0.004 |
| TG (mg/dL) | 92.00 (61.00-134.00) | 63.00 (46.00-85.50) | | | 100.00 (68.00-144.00） | ＜0.001 |
| TyG index | 8.50 (8.02-8.96) | 8.02 (7.70-8.38) | | | 8.61 (8.19-9.04) | ＜0.001 |
| ＜8.02 | 433 (25.10) | 362 (35.90) | | | 71 (9.90) |  |
| ≥8.02, ＜8.50 | 431 (25.00) | 305 (30.20) | | | 126 (17.50) |  |
| ≥8.5, ＜8.96 | 430 (24.90) | 209 (20.70) | | | 221 (30.80) |  |
| ≥8.96 | 433 (25.10) | 133 (13.20) | | | 300 (41.80) |  |
| HOMA-IR score | 2.24 (1.38-3.84) | 1.18 (0.77-1.67) | | | 2.68 (1.69-4.49) | ＜0.001 |
| ＜1.38 | 432 (25.00) | 386 (38.30) | | | 46 (6.40) |  |
| ≥1.38, ＜2.24 | 432 (25.00) | 311 (30.80) | | | 121 (16.90) |  |
| ≥2.24, ＜3.84 | 432 (25.00 ) | 214 (21.20) | | | 218 (30.40) |  |
| Variables | Total, n=1727  n (%) or M (Q1-Q3) | | non-MAFLD, n=1009  n (%) or M (Q1-Q3) | | MAFLD, n=718  n (%) or M (Q1-Q3) | *P* value^*^ |
| ≥3.84 | 431 (25.00) | | 98 (9.70) | | 333 (46.40) |  |
| TyG-WHtR | 5.05 (4.34-5.81) | | 3.86 (3.54-4.25) | | 5.32 (4.76-6.01) | ＜0.001 |
| ＜4.34 | 433 (25.10) | | 412 (40.80) | | 21 (2.90) |  |
| ≥4.34, ＜5.05 | 430 (24.90) | | 297 (29.40) | | 133 (18.50) |  |
| ≥5.05, ＜5.81 | 432 (25.00) | | 203 (20.10) | | 229 (31.90) |  |
| ≥5.81 | 432 (25.00) | | 97 (9.60) | | 335 (46.70) |  |
| TyG-BMI | 240.41 (203.68-287.39) | | 176.32 (161.74-193.57) | | 255.49 (226.35-298.87) | ＜0.001 |
| ＜203.68 | 431 (25.00） | | 41040.60) | | 21 (2.90) |  |
| ≥203.68, ＜240.41 | 432 (25.00) | | 306 (30.30) | | 126 (17.50) |  |
| ≥240.41, ＜287.39 | 432 (25.00) | | 188 (18.60) | | 244 (34.00) |  |
| ≥287.39 | 432 (25.00) | | | 105 (10.40) | 327 (45.50) |  |
| TyG-WC | 838.50 (720.71-959.81) | | | 641.76(589.18-700.32) | 879.44 (793.96-994.53) | ＜0.001 |
| ＜720.71 | 431 (25.00) | | | 410 (40.60) | 21 (2.90) |  |
| ≥720.71, ＜838.50 | 432 (25.00) | | | 301 (29.80) | 131 (18.20) |  |
| ≥838.50, ＜959.81 | 432 (25.00) | | | 200 (19.80) | 232 (32.30) |  |
| ≥959.81 | 432 (25.00) | | | 98 (9.70) | 334 (46.50) |  |

Data are presented as median and interquartile range for non-parametric variables as well as frequency and proportions for categorical variables. The characteristics of the study subjects were analyzed according to MAFLD status using the Mann-Whitney U test to compare continuous variables and the Chi-squared test for categorical variables.

^a^ variables divided by quartiles.

^b^ Overweight or obesity was defined as BMI ≥ 25 kg/m^2^.

^∗^*P* value for MAFLD and non-MAFLD group.

**Supplementary Table 2.** The optimal cut-off values of TyG-WHtR and TyG-BMI for NAFLD, MAFLD, liver fibrosis, and moderate-to-advanced fibrosis.

|  | NAFLD | | | MAFLD | | | Liver fibrosis | | | Moderate-to-advanced fibrosis | | |
| --- | --- | --- | --- | --- | --- | --- | --- | --- | --- | --- | --- | --- |
|  | TyG-WC | TyG-WHtR | TyG-BMI | TyG-WC | TyG-WHtR | TyG-BMI | TyG-WC | TyG-WHtR | TyG-BMI | TyG-WC | TyG-WHtR | TyG-BMI |
| Cut-off values | 822.34 | 4.94 | 237.77 | 822.34 | 4.94 | 237.77 | 1030.95 | 5.84 | 290.61 | 1033.35 | 5.68 | 309.08 |
| Sensitivity (%) | 80.87 | 81.68 | 79.38 | 83.01 | 83.84 | 81.48 | 56.47 | 70.00 | 65.88 | 60.91 | 82.73 | 60.00 |
| Specificity (%) | 66.87 | 65.66 | 69.09 | 67.49 | 66.30 | 69.67 | 78.31 | 64.55 | 65.61 | 76.08 | 54.86 | 73.52 |

**Supplementary Table 3.** Discriminatory accuracy and cut-off values for TyG index, HOMA-IR, TyG-WHtR, TyG-BMI, and TyG-WC; as well as the sensitivity analysis of excluding subjects with hyperlipidemia.

1. NAFLD and MAFLD

| Variables | NAFLD | | | | | | MAFLD | | | | | |
| --- | --- | --- | --- | --- | --- | --- | --- | --- | --- | --- | --- | --- |
|  | AUC | 95%CI | YI | Cut-offs | Sensitivity | Specificity | AUC | 95%CI | YI | Cut-offs | Sensitivity | Specificity |
| Original dataset, n=1727 | | |  |  |  |  |  |  |  |  |  |  |
| TyG-WC | 0.815 | 0.796-0.833 | 0.477 | >237.77 | 79.38 | 69.09 | 0.832 | 0.814-0.850 | 0.505 | >237.77 | 81.48 | 69.67 |
| TyG-WHtR | 0.809 | 0.789-0.827 | 0.473 | >4.94 | 81.68 | 65.66 | 0.826 | 0.808-0.844 | 0.502 | >4.94 | 83.84 | 66.3 |
| TyG-BMI | 0.804 | 0.784-0.822 | 0.485 | >237.77 | 79.38 | 69.09 | 0.485 | 0.803-0.840 | 0.512 | >237.77 | 81.48 | 69.67 |
| HOMA-IR | 0.788 | 0.768-0.807 | 0.456 | >2.37 | 72.59 | 73.03 | 0.805 | 0.785-0.823 | 0.481 | >2.37 | 74.51 | 73.54 |
| TyG | 0.737 | 0.716-0.758 | 0.371 | >8.55 | 67.57 | 69.49 | 0.749 | 0.728-0.769 | 0.39 | >8.55 | 69.08 | 69.87 |
| Sensitivity analysis dataset^a^, n=1007 | | |  |  |  |  |  |  |  |  |  |  |
| TyG-WC | 0.832 | 0.808-0.855 | 0.541 | >792.18 | 86.67 | 67.41 | 0.854 | 0.830-0.875 | 0.578 | >792.18 | 89.78 | 68.06 |
| TyG-WHtR | 0.826 | 0.801-0.849 | 0.517 | >4.92 | 78.4 | 73.26 | 0.847 | 0.823-0.869 | 0.55 | >4.92 | 81.22 | 73.8 |
| TyG-BMI | 0.812 | 0.787-0.836 | 0.505 | >228.54 | 82.93 | 67.56 | 0.836 | 0.811-0.858 | 0.541 | >228.53 | 85.91 | 68.22 |
| HOMA-IR | 0.785 | 0.759-0.810 | 0.443 | >2.42 | 68.53 | 75.79 | 0.807 | 0.781-0.831 | 0.473 | >2.42 | 70.99 | 76.28 |
| TyG | 0.721 | 0.692-0.748 | 0.336 | >8.36 | 63.47 | 70.09 | 0.734 | 0.706-0.761 | 0.353 | >8.37 | 64.64 | 70.7 |

1. Liver fibrosis and moderate-to-advancedfibrosis

| Variables | Liver fibrosis | | | | | | Moderate-to-advancedfibrosis | | | | | |
| --- | --- | --- | --- | --- | --- | --- | --- | --- | --- | --- | --- | --- |
|  | AUC | 95%CI | YI | Cut-offs | Sensitivity | Specificity | AUC | 95%CI | YI | Cut-offs | Sensitivity | Specificityy |
| Original dataset, n=1727 | | | |  |  |  |  |  |  |  |  |  |
| TyG-WC | 0.724 | 0.691-0.756 | 0.348 | >290.61 | 65.88 | 65.61 | 0.740 | 0.706-0.771 | 0.37 | >1033.35 | 60.91 | 76.08 |
| TyG-WHtR | 0.723 | 0.689-0.755 | 0.346 | >5.84 | 70 | 64.55 | 0.742 | 0.709-0.773 | 0.376 | >5.68 | 82.73 | 54.86 |
| TyG-BMI | 0.708 | 0.674-0.741 | 0.315 | >290.61 | 65.88 | 65.61 | 0.713 | 0.679-0.746 | 0.335 | >309.08 | 60 | 73.52 |
| HOMA-IR | 0.702 | 0.668-0.735 | 0.356 | >5.43 | 55.88 | 79.72 | 0.7 | 0.666-0.733 | 0.364 | >5.47 | 59.09 | 77.35 |
| TyG | 0.566 | 0.529-0.602 | 0.119 | >8.87 | 55.29 | 56.61 | 0.605 | 0.569-0.641 | 0.204 | >8.86 | 63.64 | 56.78 |
| Sensitivity analysis dataset^a^, n=1007 | | |  |  |  |  |  |  |  |  |  |  |
| TyG-WC | 0.729 | 0.700-0.756 | 0.393 | >880.24 | 65.25 | 74.02 | 0.776 | 0.749-0.802 | 0.432 | >879.20 | 71.26 | 71.96 |
| TyG-WHtR | 0.730 | 0.702-0.757 | 0.405 | >5.33 | 65.25 | 75.29 | 0.777 | 0.750-0.803 | 0.478 | >5.62 | 65.52 | 82.28 |
| TyG-BMI | 0.708 | 0.678-0.735 | 0.363 | >246.99 | 70.21 | 66.05 | 0.759 | 0.731-0.785 | 0.422 | >269.69 | 64.37 | 77.83 |
| HOMA-IR | 0.695 | 0.665-0.723 | 0.327 | >3.71 | 51.06 | 81.64 | 0.713 | 0.684-0.741 | 0.391 | >4.90 | 50.57 | 88.48 |
| TyG | 0.624 | 0.593-0.654 | 0.212 | >8.38 | 59.57 | 61.66 | 0.672 | 0.642-0.701 | 0.283 | >8.73 | 45.98 | 82.28 |

^a^ Sensitivity analysis was performed with 1007 individuals excluding subjects with hyperlipidemia (defined as high

levels of fast-ing serum cholesterol (>200 mg/dl) or high triglycerides (>150 mg/dl).

**Supplementary Table 4.** Comparison of the predictive capabilities of TyG-WHtR and TyG-BMI in NAFLD and MAFLD among different subgroups.

1. The subgroup analyses for NAFLD

|  | TyG-WHtR  AUC (95%CI) | *P* value | TyG-BMI  AUC (95%CI) | *P* value |
| --- | --- | --- | --- | --- |
| Age ≤ 53 | 0.771 (0.742-0.799) | 0.039 | 0.825 (0.799-0.850) | 0.007 |
| Age > 53 | 0.829 (0.802-0.853) |  | 0.782 (0.753-0.809) |  |

1. The subgroup analyses for MAFLD

|  | TyG-WHtR  AUC (95%CI) | *P* value | TyG-BMI  AUC (95%CI) | *P* value |
| --- | --- | --- | --- | --- |
| Age≤53 | 0.854 (0.828 - 0.877) | <0.001 | 0.852 (0.826 - 0.875) | 0.004 |
| Age>53 | 0.784 (0.755 - 0.811) |  | 0.795 (0.766 - 0.821) |  |
| Non-diabetes | 0.816 (0.794 - 0.836) | 0.031 | 0.818 (0.796 - 0.838) | 0.026 |
| Diabetes | 0.751 (0.706 - 0.792) |  | 0.751 (0.705 - 0.792) |  |
| Non-overweight /obesity | 0.854 (0.820 - 0.884) | <0.001 | 0.832 (0.796 - 0.863) | 0.002 |
| Overweight /obesity | 0.745 (0.719 - 0.769) |  | 0.734 (0.708 - 0.758) |  |

Note: Only the statistically significant (*P* < 0.05) results of subgroups are shown.
