# Supplementary figures and images for: Potential screening indicators for early diagnosis of NAFLD/MAFLD and liver fibrosis: Triglyceride glucose index–related parameters
Source: Front Endocrinol (Lausanne). 2022 Sep 2;13:951689. doi: 10.3389/fendo.2022.951689 (PMC9478620; doi:10.3389/fendo.2022.951689)

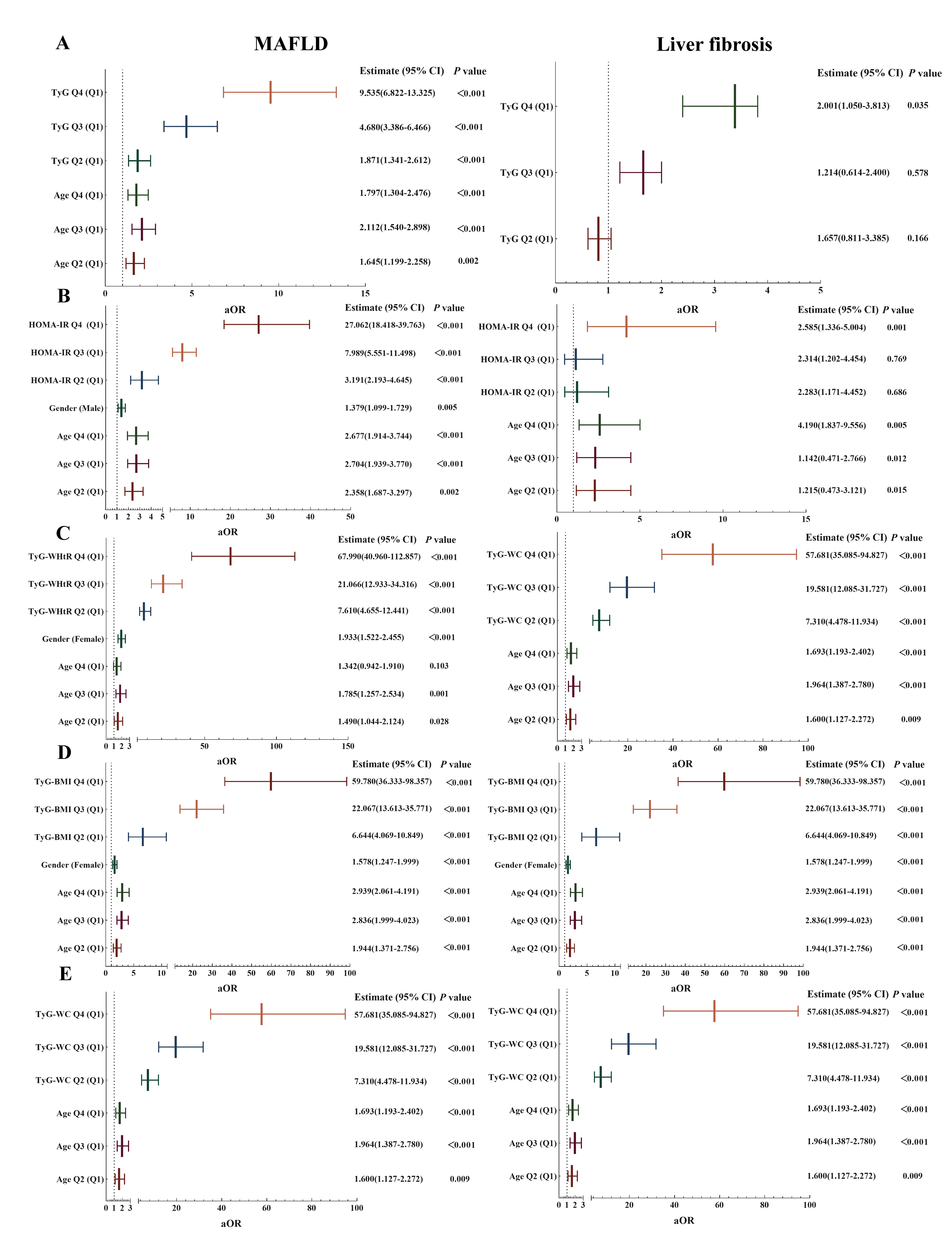

Supplement: Supplementary file 2 [file Image_1.tif]

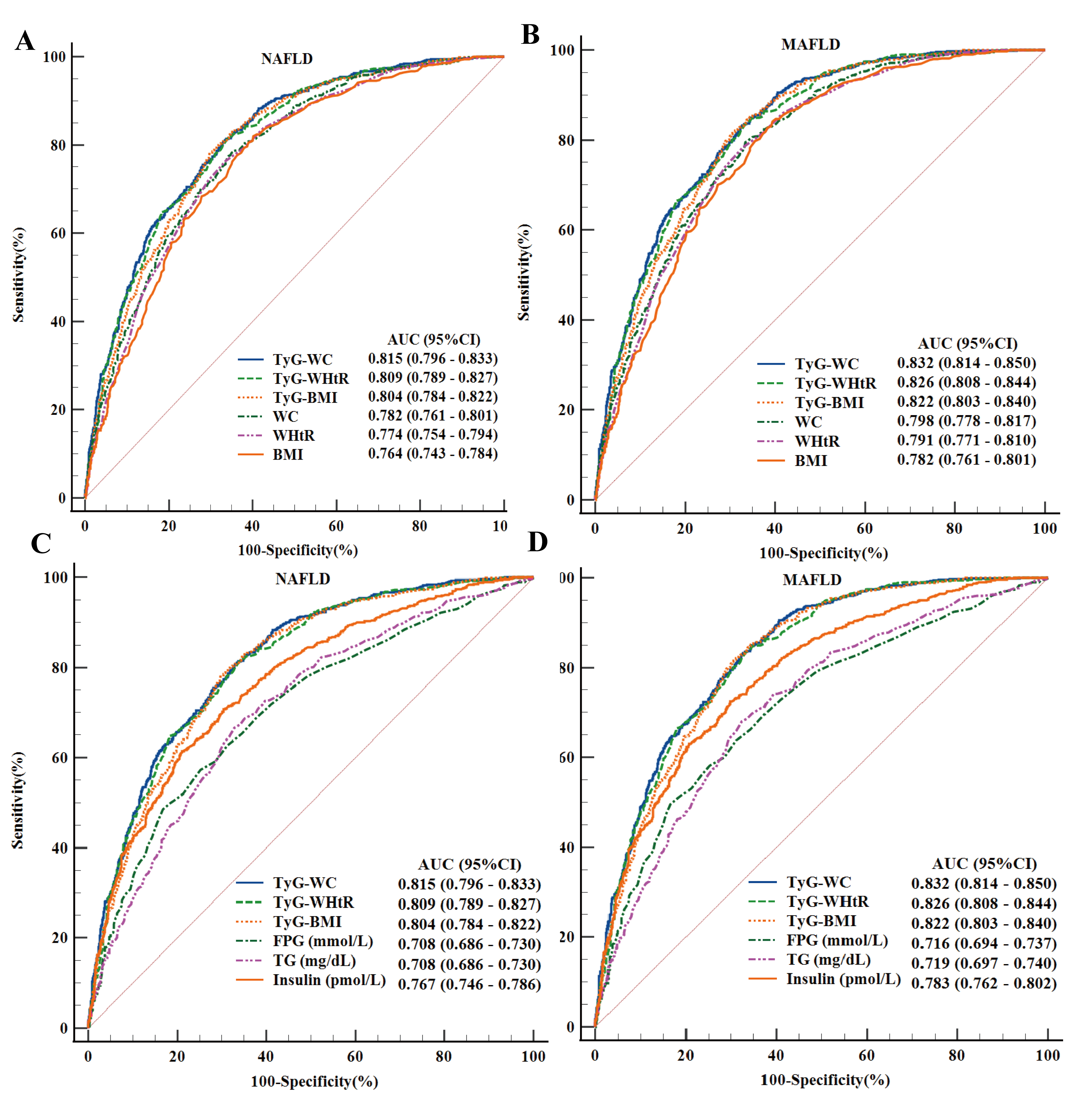

Supplement: Supplementary file 3 [file Image_2.tif]

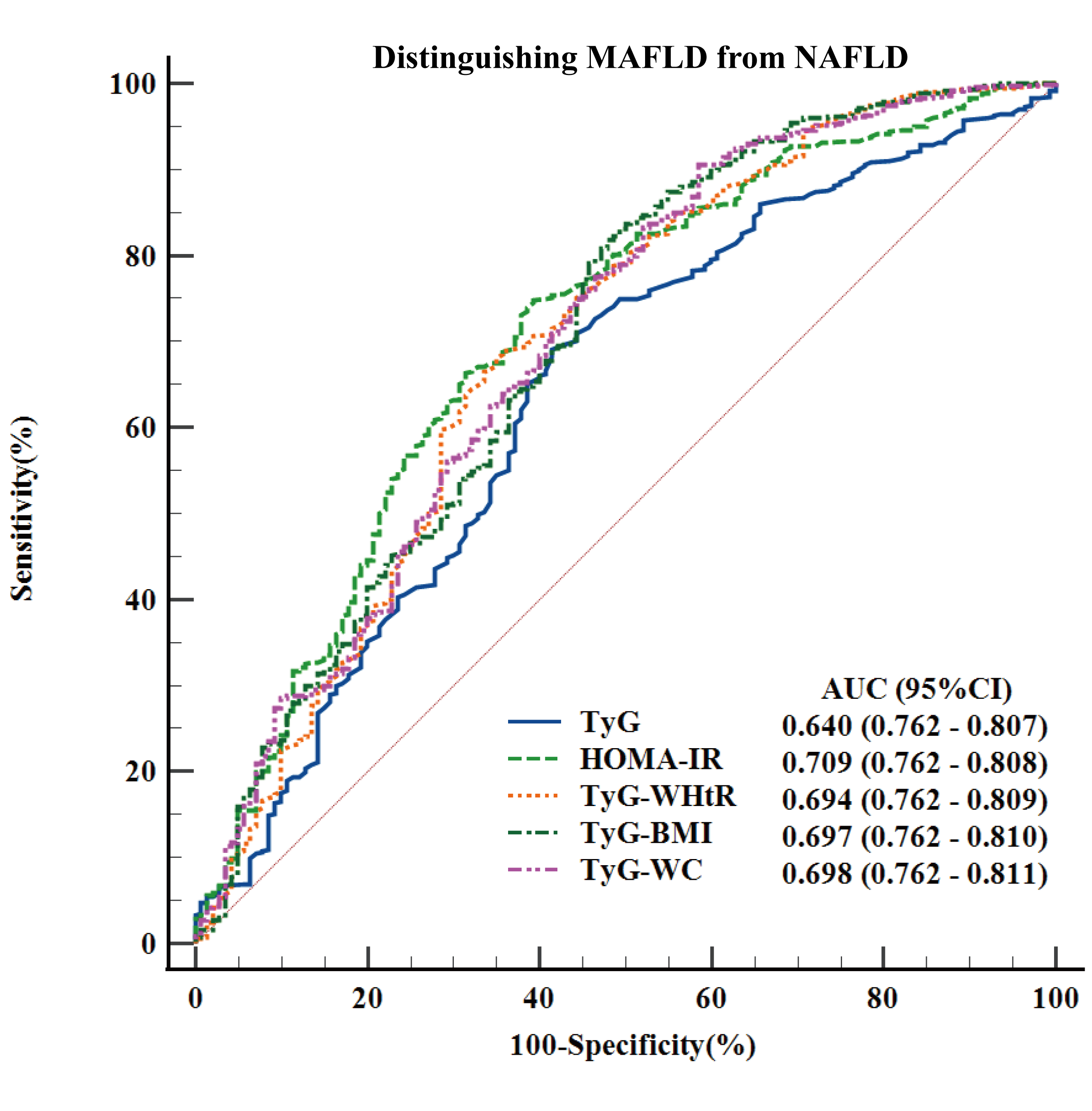

Supplement: Supplementary file 4 [file Image_3.tif]

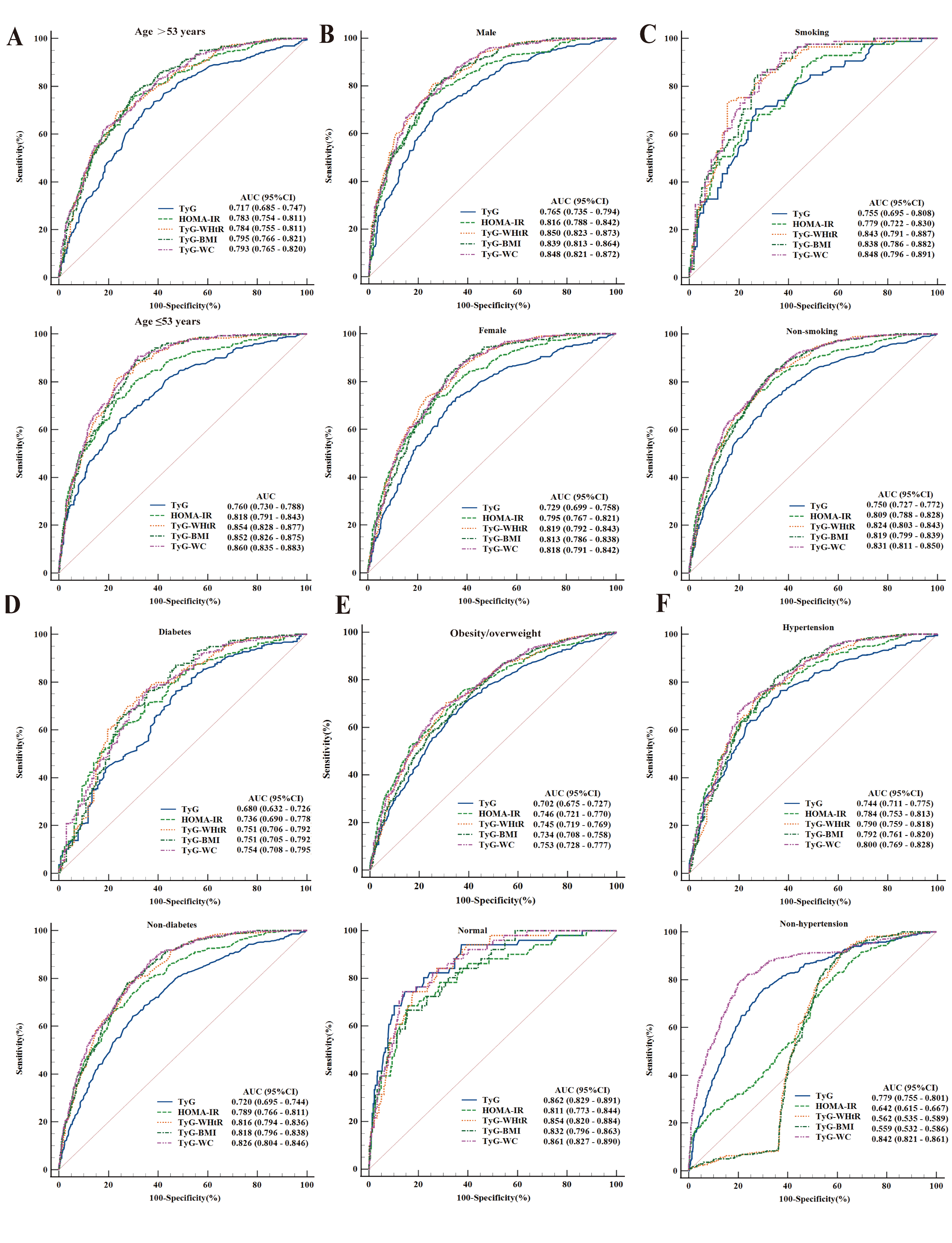

Supplement: Supplementary file 5 [file Image_4.tif]

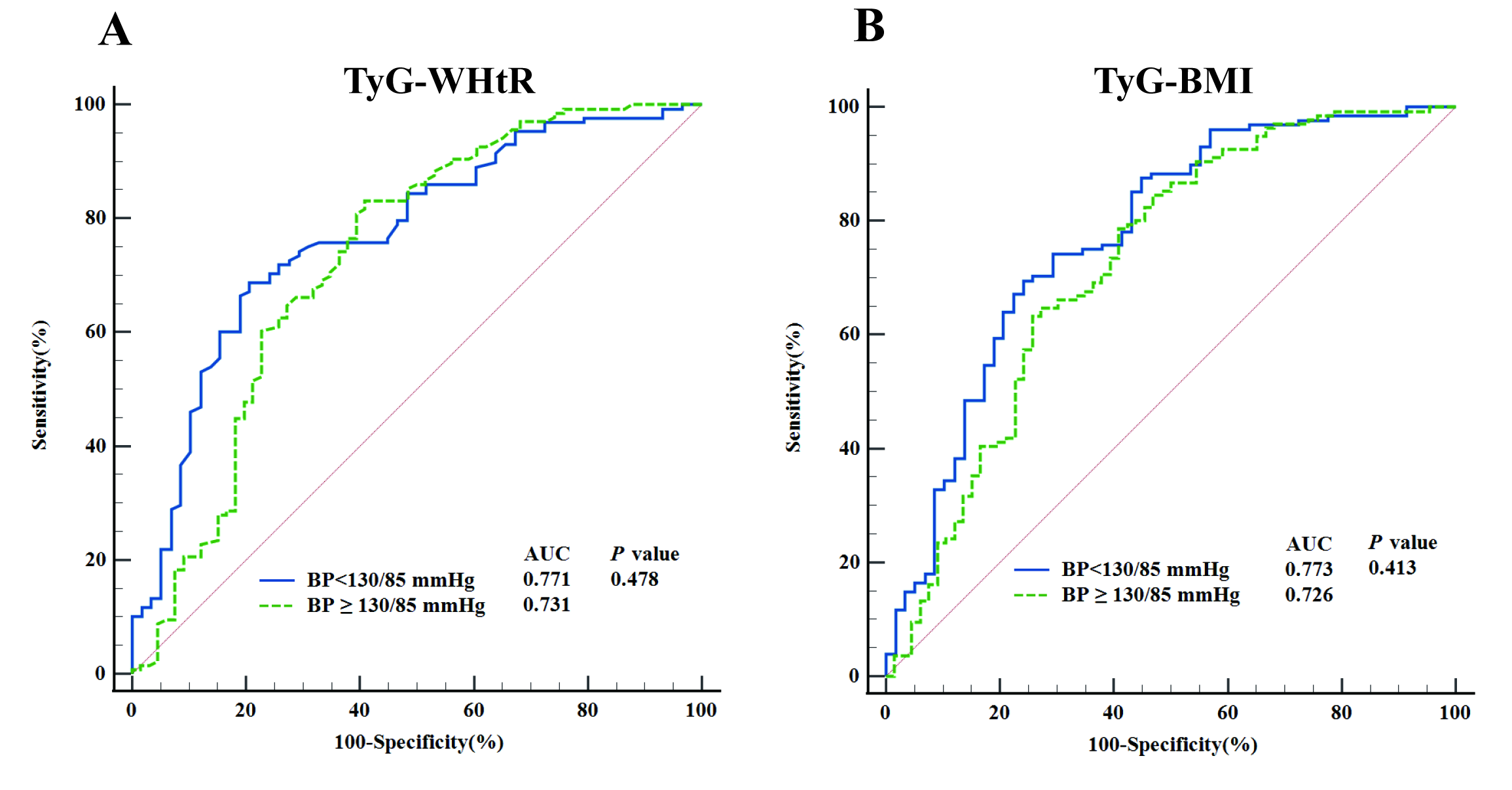

Supplement: Supplementary file 6 [file Image_5.tif]

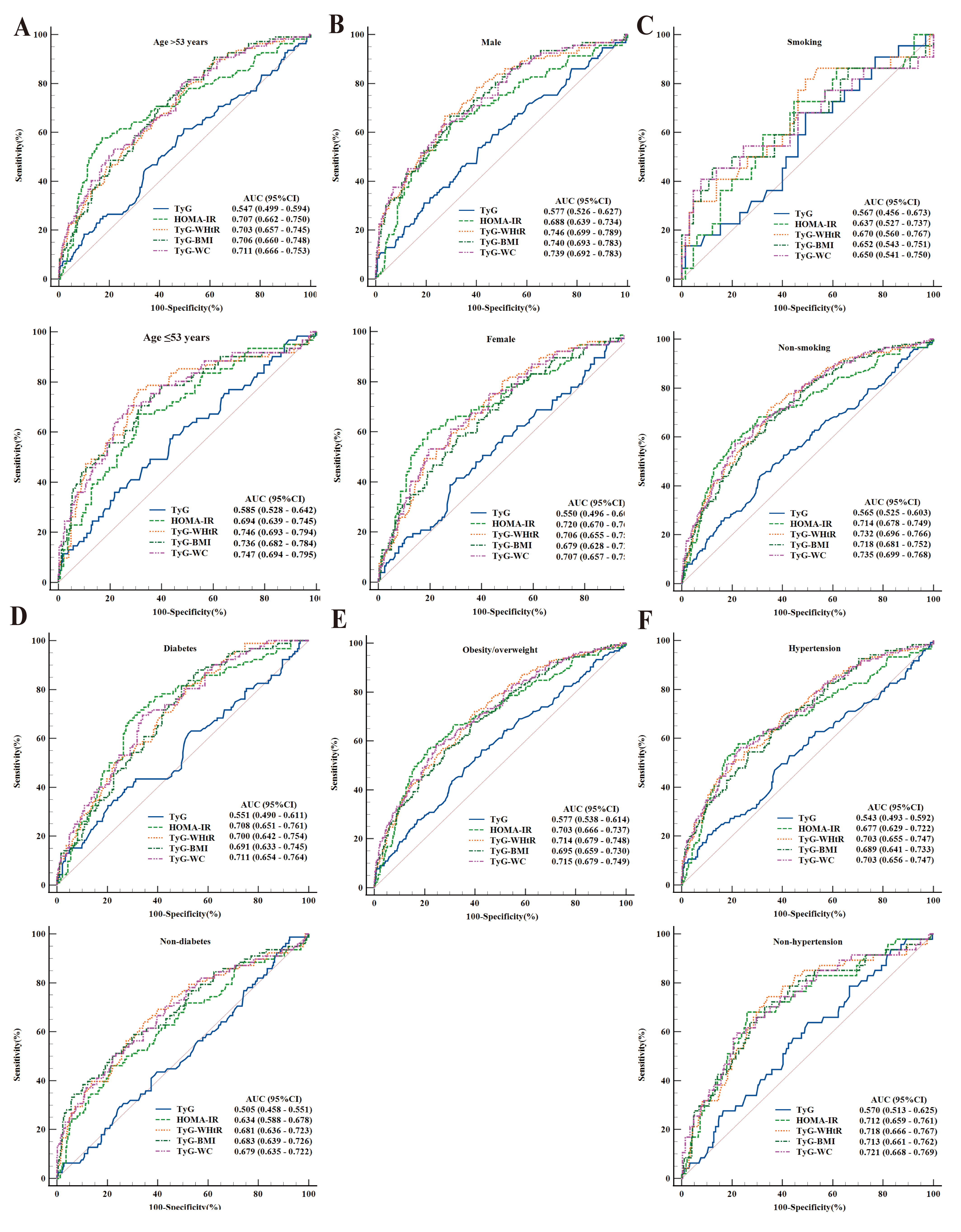

Supplement: Supplementary file 7 [file Image_6.tif]

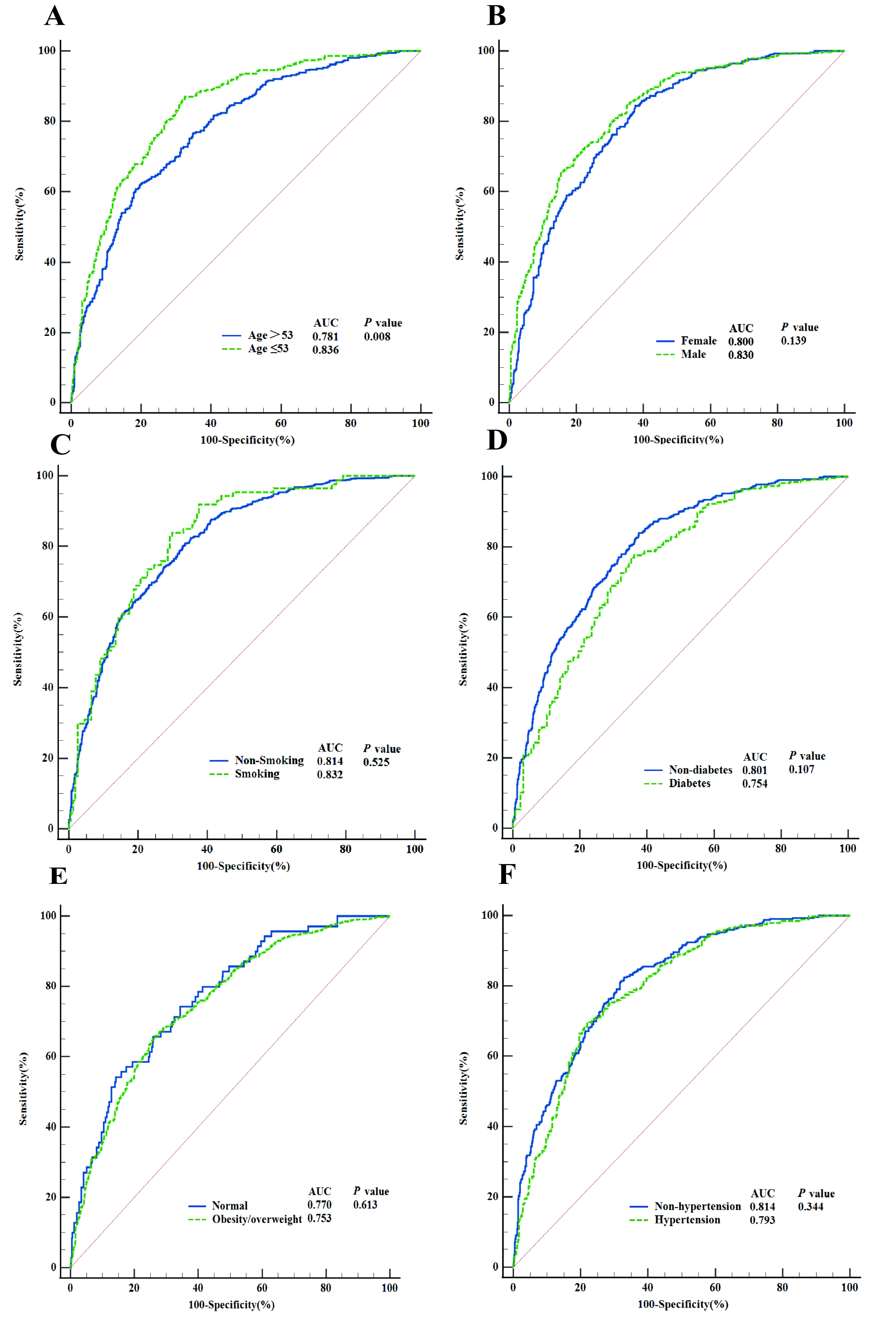

Supplement: Supplementary file 8 [file Image_7.tif]

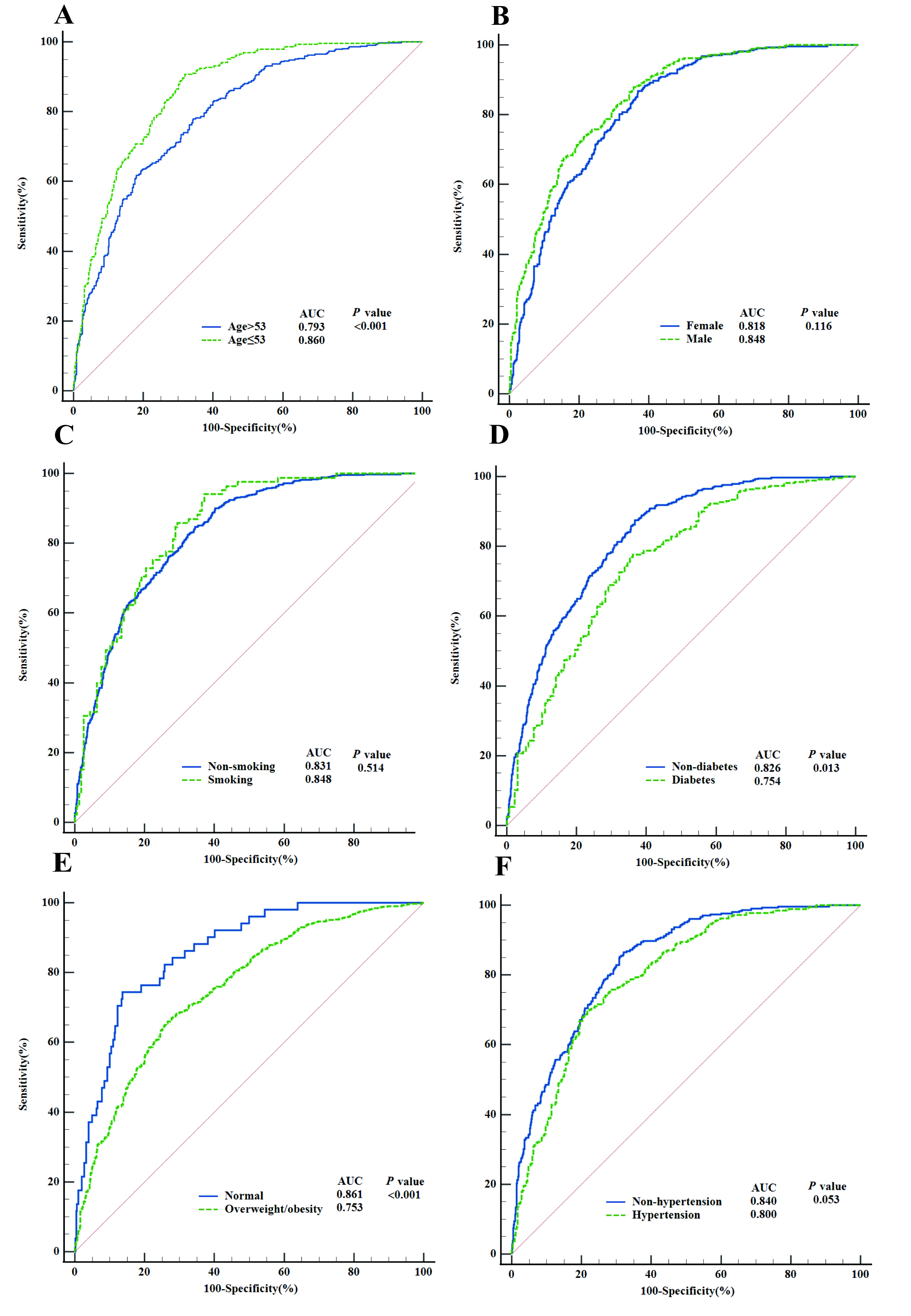

Supplement: Supplementary file 9 [file Image_8.tif]

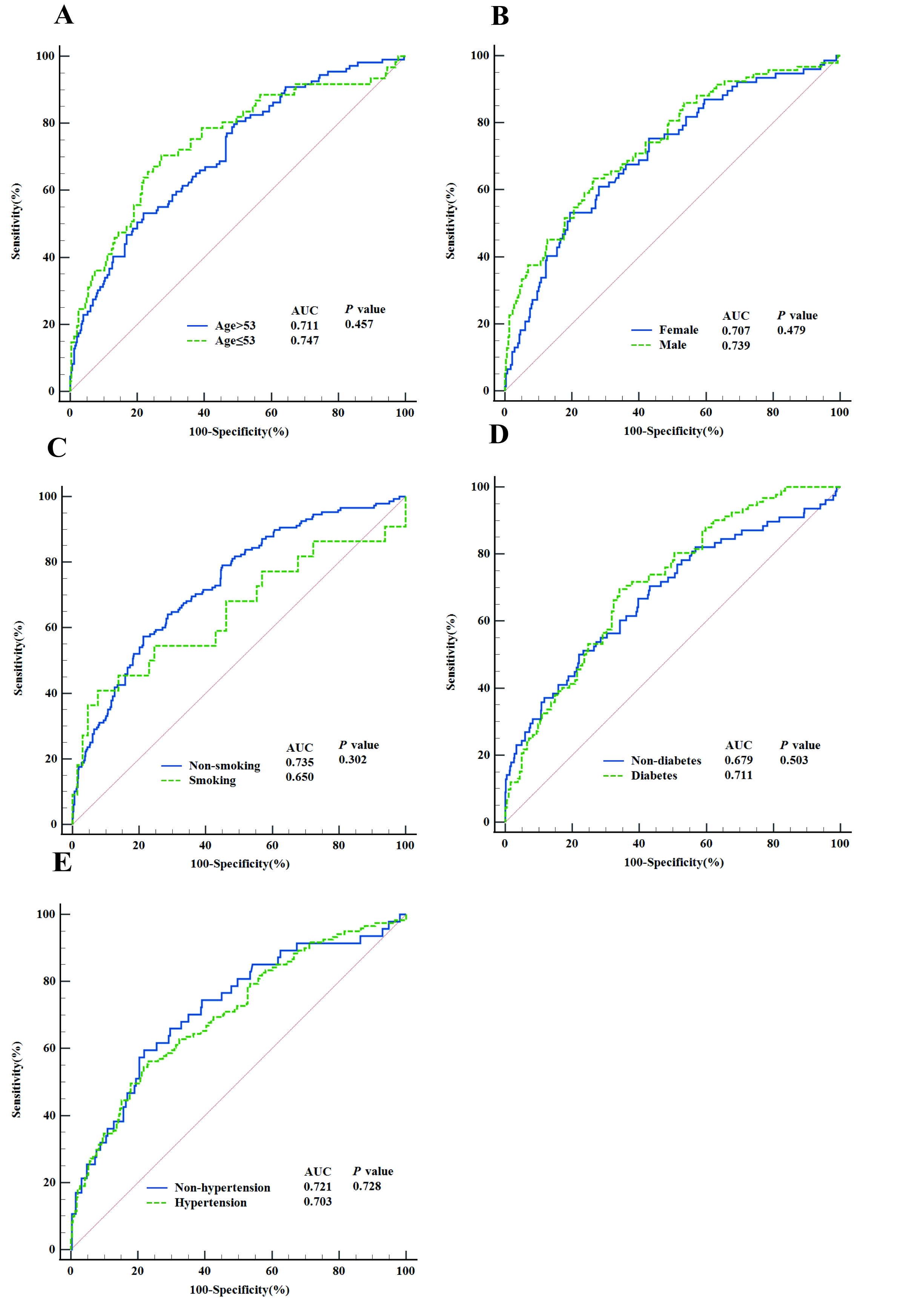

Supplement: Supplementary file 10 [file Image_9.tif]
